# PD-L1 tumor-intrinsic signaling and its therapeutic implication in triple-negative breast cancer

**DOI:** 10.1172/jci.insight.131458

**Published:** 2021-04-22

**Authors:** Chunhua Chen, Shiheng Li, Junli Xue, Manlong Qi, Xin Liu, Yan Huang, Jinghua Hu, Haidong Dong, Kun Ling

**Affiliations:** 1 Department of Biochemistry and Molecular Biology,; 2 Departments of Urology and Immunology, and; 3 Department of Nephrology and Hypertension, Mayo Clinic, Rochester, Minnesota, USA.

**Keywords:** Oncology, Therapeutics, Breast cancer, Immunotherapy, Ubiquitin-proteosome system

## Abstract

Although the immune checkpoint role of programmed death ligand 1 (PD-L1) has been established and targeted in cancer immunotherapy, the tumor-intrinsic role of PD-L1 is less appreciated in tumor biology and therapeutics development, partly because of the incomplete mechanistic understanding. Here we demonstrate a potentially novel mechanism by which PD-L1 promotes the epithelial-mesenchymal transition (EMT) in triple-negative breast cancer (TNBC) cells by suppressing the destruction of the EMT transcription factor Snail. PD-L1 directly binds to and inhibits the tyrosine phosphatase PTP1B, thus preserving p38-MAPK activity that phosphorylates and inhibits glycogen synthase kinase 3β (GSK3β). Via this mechanism, PD-L1 prevents the GSK3β-mediated phosphorylation, ubiquitination, and degradation of Snail and consequently promotes the EMT and metastatic potential of TNBC. Significantly, PD-L1 antibodies that confine the tumor-intrinsic PD-L1/Snail pathway restricted TNBC progression in immunodeficient mice. More importantly, targeting both tumor-intrinsic and tumor-extrinsic functions of PD-L1 showed strong synergistic tumor suppression effect in an immunocompetent TNBC mouse model. Our findings support that PD-L1 intrinsically facilitates TNBC progression by promoting the EMT, and this potentially novel PD-L1 signaling pathway could be targeted for better clinical management of PD-L1–overexpressing TNBCs.

## Introduction

Cancer cells often upregulate programmed death ligand 1 (PD-L1) to exhaust activated T cells by stimulating PD-1 on T cells (1, 2). Thus, antibodies that block PD-1/PD-L1 binding have been applied in clinical trials to prevent immune evasion in various types of cancers (3). Recent clinical trials confirmed promising outcomes but also exposed imperfections (low response rate, side effects, and resistance) that indicate the incomplete understanding of the PD-L1 pathway. Triple-negative breast cancer (TNBC) remains a major clinical challenge with the worst outcome of all BC subtypes, mostly because of its poor response to current therapies and high incidence of metastasis. Since TNBC cells express higher levels of PD-L1 more often than other BC subtypes (4), PD-L1 antibodies, as monotherapy or a part of combined therapies, have been applied in multiple clinical trials for cancers including TNBCs (5). Current results from these trials suggest that the combined therapy yields synergistic effects (5), which endorses the FDA approval of atezolizumab (a PD-L1 antibody) combined with paclitaxel for PD-L1–positive TNBC (6). However, the rational combination and successful application of anti–PD-L1 therapy are dependent upon the comprehensive understanding of PD-L1 biology in tumor cells.

In addition to its tumor-extrinsic role of activating PD-1 on immune cells, PD-L1 may influence cancer progression by regulating various tumor-intrinsic events in tumor cells independent of the immune system (7, 8). For example, an association of PD-L1 and the epithelial-mesenchymal transition (EMT) was observed in clinical studies of various cancers (9–14) as well as in PD-L1–transgenic mice (15, 16). Studies in claudin-low breast cancer (17) and cervical cancer (18) also suggested that PD-L1 promotes cancer aggressiveness by influencing the tumor-intrinsic signaling events in the EMT, metabolism, and metastasis. Considering the significant role of the EMT in various aspects of tumor progression, including growth, metastasis, stemness, treatment resistance, dormancy (19), the connection of PD-L1 with the EMT in tumor cells shed light on novel avenues for understanding tumor biology. However, these studies are mostly descriptive, and the molecular mechanism connecting PD-L1 to the EMT in TNBC cells remains unclear. In particular, the biological significance and clinical indication of PD-L1’s tumor-intrinsic functions are vague.

Here, we demonstrate that PD-L1 facilitates the EMT in TNBC cells by protecting the EMT-promoting transcription factor Snail from being destructed by the proteosome. This tumor-intrinsic function of PD-L1, which is achieved via the protein tyrosine phosphatase1B (PTP1B)/p38-MAPK/glycogen synthase kinase 3β (GSK3β) axis and can be activated by PD-1 binding, regulates the aggressiveness of TNBC cells. Intriguingly, PD-L1 antibodies that constrain its tumor-intrinsic pathway inhibited the growth and metastasis of TNBC tumors in immunodeficient mice. More importantly, blockade of both tumor-extrinsic and -intrinsic functions of PD-L1 synergistically suppressed cancer progression in a syngeneic, immunocompetent TNBC mouse model. These results broaden the current paradigm regarding the role of PD-L1 in cancer progression and emphasize an underestimated concept that the tumor-intrinsic PD-L1 pathway needs to be considered when applying the anti–PD-L1 therapy.

## Results

### Expression of PD-L1 intrinsically promotes the EMT and aggressive malignancy of TNBC cells.

**Figure 1:**
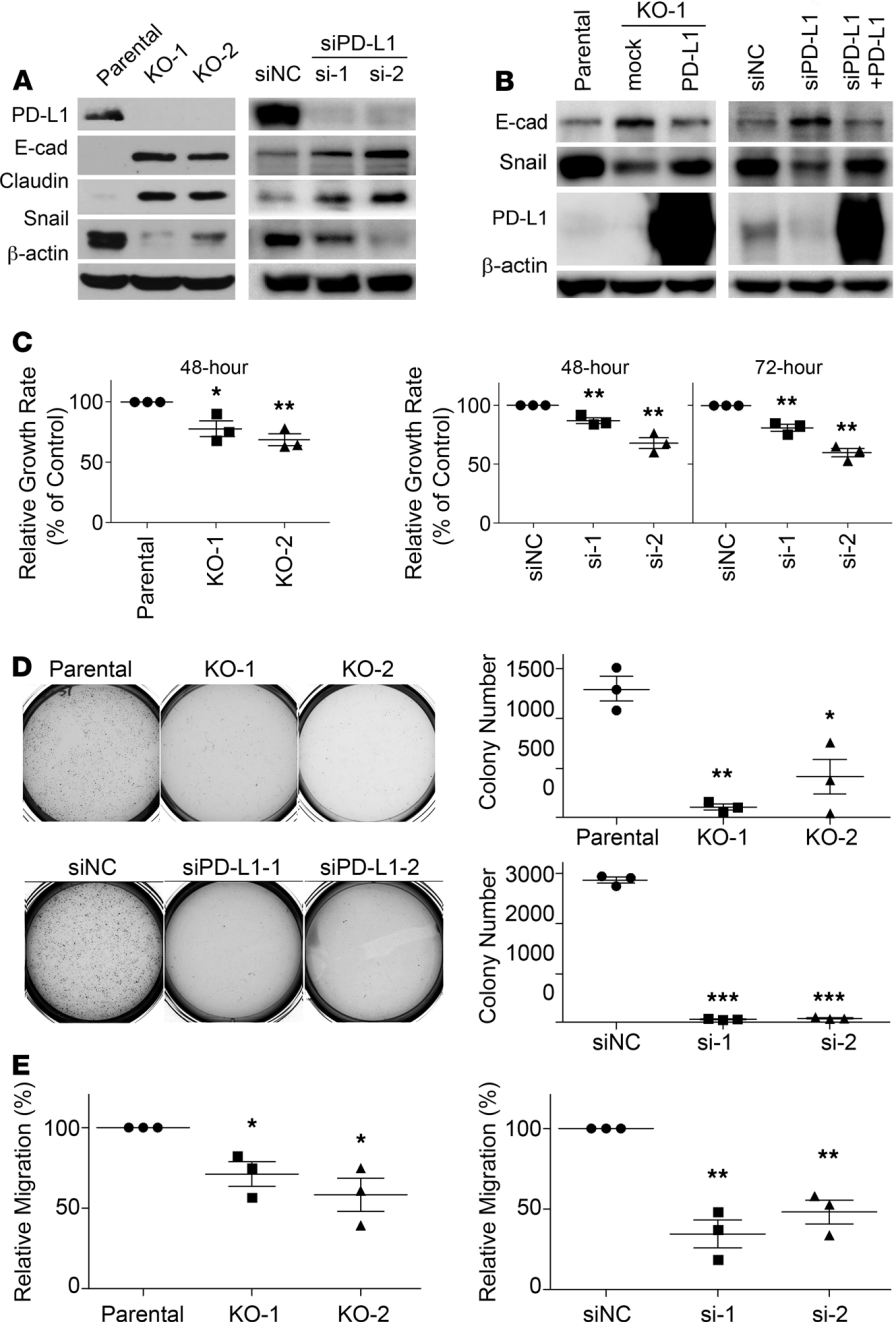
Expression of PD-L1 promotes the EMT and aggressive behaviors in MDA-MB-231 cells. (**A**) Loss of PD-L1 induced epithelial characteristics in TNBC cells. Left panels, cell lysates from the parental or 2 clones of PD-L1–null (KO-1 and KO-2) MDA-MB-231 cells. Right panels, MDA-MB-231 cells transiently transfected with nonspecific control siRNA (siNC) or 2 distinct PD-L1 specific (siPD-L1) siRNAs (si-1 and si-2) for 48 hours. (**B**) Reexpression of PD-L1 in PD-L1–deficient MDA-MB-231 cells restored the expression of E-cadherin and Snail to levels comparable to the parental cells. Cells were transfected with PD-L1 for 24 hours followed by siPD-L1 (si-1) for another 48 hours. (**C**) PD-L1 deficiency decreased cell proliferation. Cell proliferation of the parental or PD-L1–deficient cells was determined at 48 hours or 72 hours. (**D**) Loss of PD-L1 inhibited the anchorage-independent growth. Tumorigenesis potential of control or PD-L1–deficient MDA-MB-231 cells was determined using soft agar colony formation assay. Colony numbers were counted using GelCount. (**E**) Cells lacking PD-L1 were less migratory. The in vitro cell migration assay was performed using Boyden chamber with 20,000 cells/chamber. Directional cell migration was induced by a 4-hour treatment of 10% FBS in cells serum-starved overnight. (**C**–**E**) Results (*n* = 3 independent experiments) were statistically analyzed and plotted as mean ± SEM using unpaired 2-tailed Student’s *t* test with the *P* value adjusted by Bonferroni’s method. *, *P* < 0.05; **, *P* < 0.01; ***, *P* < 0.001.

To understand PD-L1 function in TNBC cells, we employed human TNBC cell line MDA-MB-231 that exhibits high PD-L1 expression (4). Utilizing the CRISPR/Cas9 system, we interrupted PD-L1 expression in MDA-MB-231 cells (Figure 1A and Supplemental Figure 1, A and B; supplemental material available online with this article; https://doi.org/10.1172/jci.insight.131458DS1). Unexpectedly, these PD-L1–null cells exhibited morphological changes and appeared epithelial like (Supplemental Figure 1C). Results from further investigation showed that protein levels of epithelial markers E-cadherin and Claudin-1 were notably increased in PD-L1–null clones compared with their WT counterparts (Figure 1A). Correspondingly, PD-L1–null cells exhibited a substantial decrease of Snail (Figure 1A), an EMT-promoting transcription factor (EMT-TF) that suppresses the expression of E-cadherin and Claudin-1 (20). To confirm this observation, we used RNA interference to achieve transient depletion of PD-L1 in MDA-MB-231 cells using 2 distinct PD-L1–specific siRNAs, which resulted in a substantial increase of E-cadherin and Claudin-1 and strongly reduced Snail (Figure 1A). Slug, a Snail family EMT-TF, also showed reduction in both PD-L1–null and PD-L1–knockdown cells (Supplemental Figure 2A), whereas another EMT-TF, zinc finger E-box binding homeobox 1 (ZEB1), was not affected by PD-L1 deficiency (Supplemental Figure 2A). Notably, suppressing PD-L1 expression in MDA-MB-231 cells led to a downregulation of matrix protein fibronectin, which is often upregulated in mesenchymal and metastatic tumor cells (21). Yet, additional mesenchymal markers, like vimentin and β-catenin, were not markedly or consistently altered, and N-cadherin remained unexpressed when PD-L1 expression was blocked in MDA-MB-231 cells (Supplemental Figure 2A). These results suggest that loss of PD-L1 partially reversed the EMT status in MDA-MB-231 cells. Importantly, reexpression of PD-L1 (Supplemental Figure 2B) in both PD-L1–null and PD-L1–knockdown MDA-MB-231 cells overwhelmed the E-cadherin expression and restored Snail (Figure 1B). Thus, we reason that PD-L1 promotes the EMT in MDA-MB-231 cells, which is likely caused by upregulating Snail family TFs.

To determine if this phenomenon is cell line specific, we examined another PD-L1–expressing human TNBC cell line, Hs578T. As in MDA-MB-231 cells, depletion of PD-L1 in Hs578T cells caused a marked decrease of Snail but appeared to have no effect on Slug and ZEB1 (Supplemental Figure 3, A and B). Correspondingly, typical mesenchymal markers, including N-cadherin, fibronectin, and β-catenin, were decreased in PD-L1–depleted Hs578T cells (Supplemental Figure 3C). Although we did not detect significant changes on expression of other examined epithelial proteins, such as E-cadherin, Claudin-1, and ZO-1 (Supplemental Figure 3D), decreases of Snail and mesenchymal markers in PD-L1–depleted Hs578T cells also suggest a partial reverse of the EMT. Thus, our results suggest that PD-L1 expression intrinsically promotes the EMT in TNBC cells.

The EMT is a transdifferentiation program that plays an important role in promoting all aspects of cancer aggressiveness and progression, including tumorigenesis, metastasis formation, resistance to apoptotic stimuli, as well as the entrance into cancer stem cell states (19, 22). To determine whether the intrinsic function of PD-L1 affects the aggressiveness of PD-L1–expressing TNBC tumors, we first determined in vitro behaviors of parental and PD-L1–deficient MDA-MB-231 cells. Compared with parental cells, MDA-MB-231 cells with stable/complete or transient/partial loss of PD-L1 showed a moderate yet significant decrease in cell growth/survival in vitro (Figure 1C). Notably, a severely weakened ability of forming tumor spheroid in soft agar was observed in PD-L1–deficient cells (Figure 1D), which could hardly survive in soft agar. These results suggest that PD-L1 plays a critical role in cell growth and MDA-MB-231 cells require PD-L1 for anchorage-independent proliferation and/or survival.

Consistent with the long-standing role of the EMT in promoting cell migration, PD-L1 deficiency inhibited the in vitro migration of MDA-MB-231 cells. The relative migration rate of the 2 PD-L1–null clones in response to FBS decreased to 65% and 52% of the parental cells, respectively (Figure 1E, left). RNA interference–mediated (RNAi-mediated) PD-L1 knockdown also substantially suppressed cell migration of MDA-MB-231 cells (Figure 1E, right). We hereby conclude that protein-level changes of epithelial markers and EMT-TFs in PD-L1–deficient tumor cells truly decreased their aggressive behaviors.

### PD-L1 depletion attenuates the lung metastasis of TNBC cells in an immunodeficient host.

**Figure 2:**
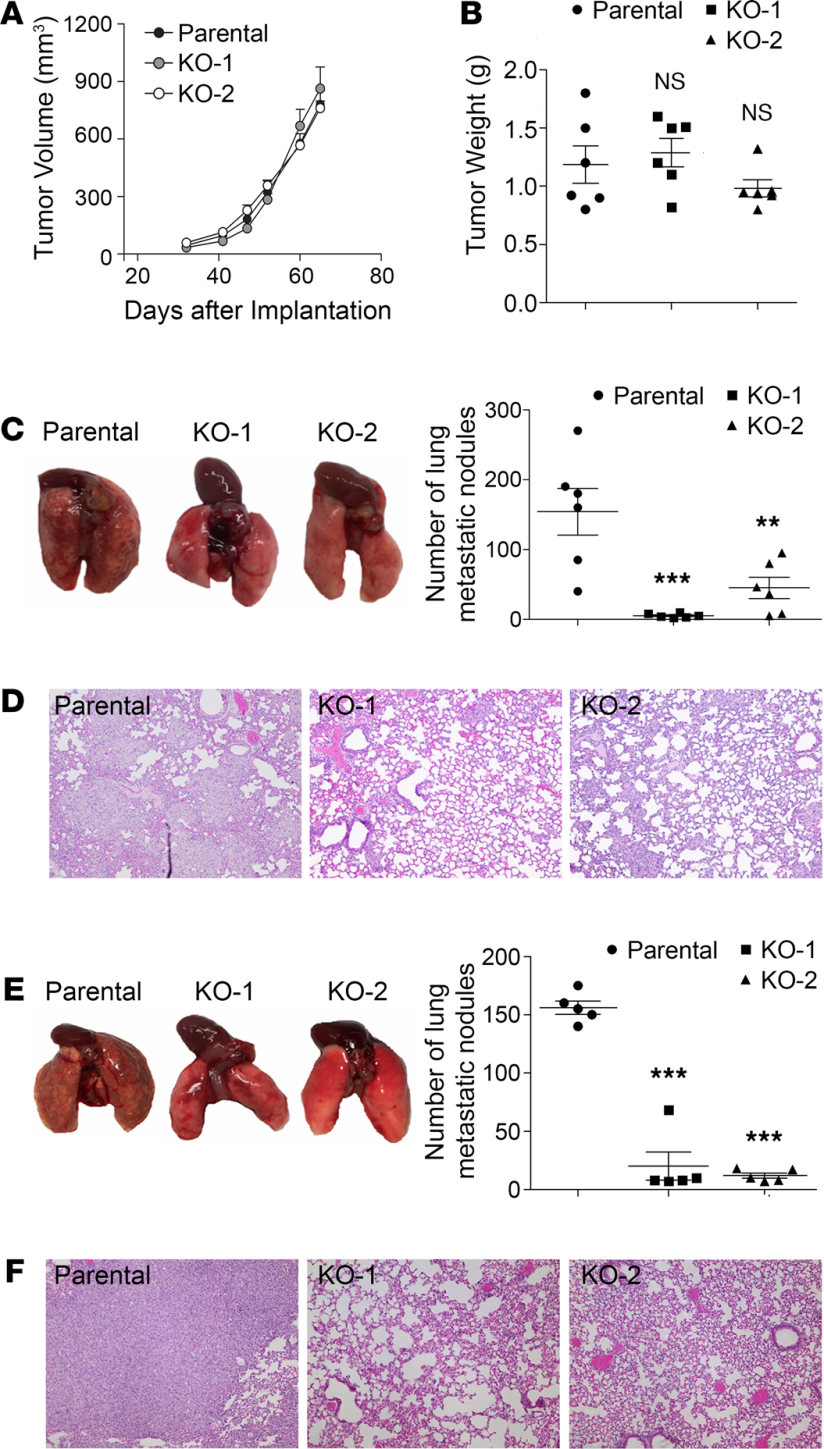
PD-L1 deficiency reduces the tumor metastasis independent of the antitumor immunity. (**A**–**D**) Two million parental or PD-L1–null (KO-1 and KO-2) cells were injected into the mammary fat pad of NOD/SCID mouse (6 mice/group). (**A**) Tumor volume was measured with calipers weekly and calculated using the standard formula. (**B**) Tumors were dissected and weighed at the endpoint (65 days after inoculation). (**C** and **D**) Loss of PD-L1 inhibits lung metastasis. (**C**) Lungs were dissected at the endpoint. Left panel, images of gross lung showed that parental MDA-MB-231 tumors generated many more metastatic nodules on lung surface. (**D**) Representative H&E staining images of lung tissues showing micrometastatic lesions from mice bearing parental tumors are much more severe than those bearing PD-L1–null tumors. (**E** and **F**) Results from experimental metastasis model suggest that PD-L1 is necessary for the later steps of metastasis formation. Parental or PD-L1–null MDA-MB-231 cells (8 × 10^5^) were directly injected into the tail vein of NOD/SCID mice (5 mice/group). Animals were terminated 40 days later to examine lung metastasis. (**E**) Metastatic nodules on lung surface in each group were analyzed. (**F**) Representative H&E staining images of lung tissues. PD-L1–null MDA-MB-231 tumors generated many fewer lung micrometastatic lesions than parental tumors. (**D** and **F**) Power of eyepiece: 10×; power of objective: 10×. (**A**–**C** and **E**) Data were plotted as mean ± SEM and statistically analyzed using 1-way ANOVA analysis with Dunnett’s test. *n* = 6 (**A**–**C**) and *n* = 5 (**E**). N.S., no significant difference; **, *P* < 0.01; ***, *P* < 0.001.

Our results from in vitro studies suggested that the tumor-intrinsic function of PD-L1 could contribute to the aggressiveness of TNBC tumors. To test this possibility, we examined the effect of PD-L1 depletion on tumor growth and metastasis in vivo. To eliminate the effect of immune response, we employed immunodeficient NOD/SCID mice as host for the orthotopic transplantation of MDA-MB-231 cells. After inoculating parental or PD-L1–null MDA-MB-231 cells into the mammary fat pad of NOD/SCID mice, we monitored the primary tumor growth by measuring tumor size weekly and scaling tumor weight at the experimental endpoint. Both PD-L1–null clones showed similar tumor growth kinetics (Figure 2A) and final tumor weight (Figure 2B) as parental MDA-MB-231 tumors, suggesting that PD-L1 deficiency did not impact the in situ growth of primary MDA-MB-231 tumors. However, the number of lung surface metastatic nodules in mice bearing PD-L1–null tumors was dramatically reduced compared with that in mice bearing parental MDA-MB-231 tumors (Figure 2C). Histological study on lung tissue sections revealed many fewer micrometastatic lesions in animals receiving PD-L1–null cells than those receiving parental cells (Figure 2D). Because the primary tumor size was comparable between the control and PD-L1–null groups, these results suggest a true suppression on metastasis that resulted from PD-L1 deficiency. This is likely caused by the loss of tumor-intrinsic functions of PD-L1 and is independent of immune checkpoint blockade, as the tumor-hosting animals lack T cells and the systemic immune response.

Metastasis is a multistep process including local spreading/invasion, intravasation, survival in the circulation, extravasation, and colonization and proliferation in the distal organ (23). To check how PD-L1 may participate in the development of metastases, we inoculated tumor cells into the tail vein of NOD/SCID mice to skip in situ early steps of metastasis, such as local invasion and intravasation. As shown in Figure 2E, visible metastatic nodules on the lung surface of control mice were evidently greater in number and size than those bearing PD-L1–null tumors. Histological investigation of lung sections confirmed that micrometastatic lesions in lung were significantly fewer when PD-L1 expression was depleted (Figure 2F). Considering our earlier finding that PD-L1 deficiency strongly inhibited the anchorage-independent growth of MDA-MB-231 cells (Figure 1D), current results suggest that PD-L1 depletion may impair the survival of circulating tumor cells in bloodstream, in addition to blocking cell migration (Figure 1E) that mainly impairs the local invasion of PD-L1–null tumors. Taken together, these results strongly suggest that PD-L1 mediates a tumor-intrinsic, tumor-promoting function and is important for TNBC metastasis in vivo.

### PD-L1 expression protects Snail proteins from being ubiquitinated and destructed.

**Figure 3:**
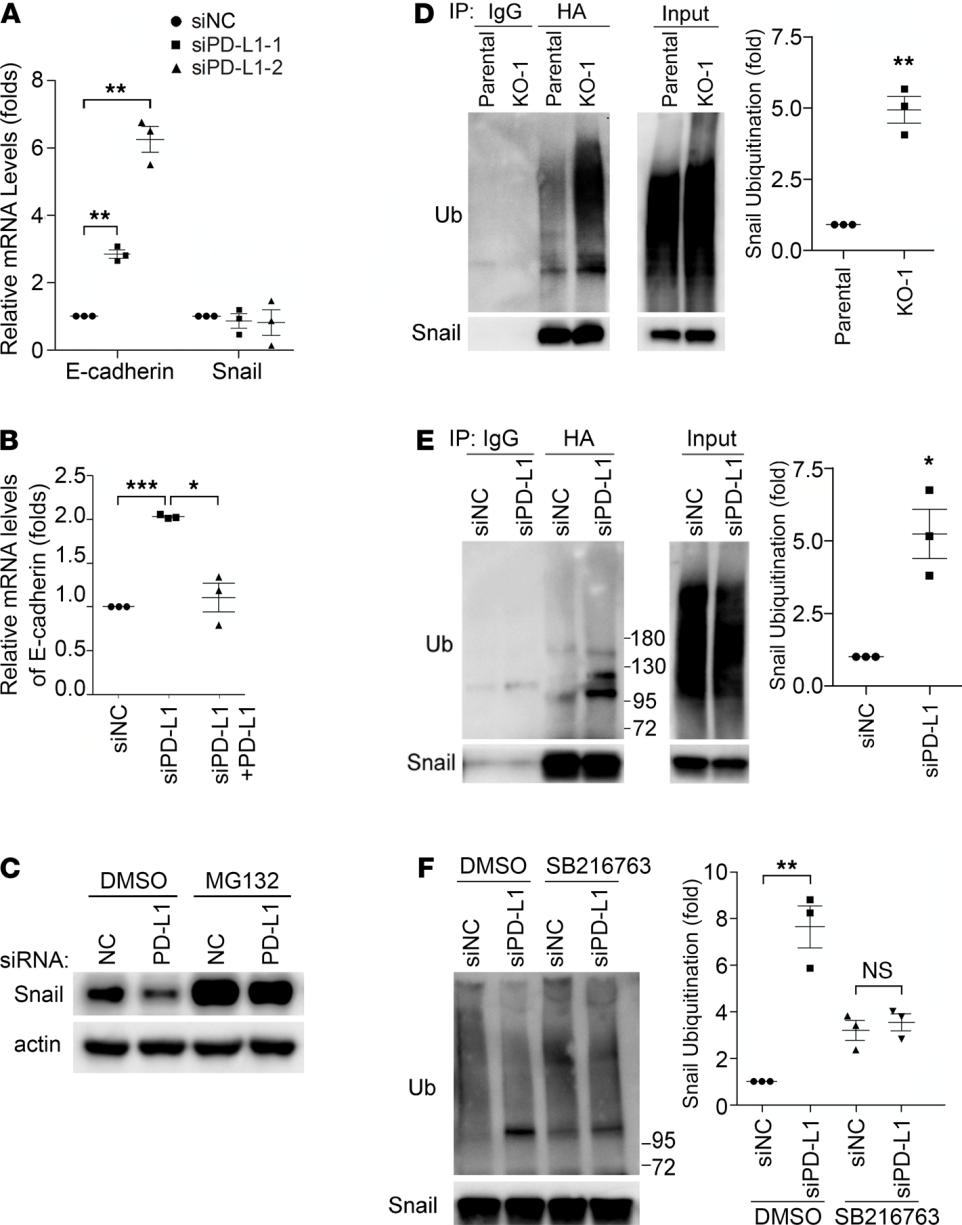
PD-L1 promotes the EMT by protecting Snail from being ubiquitinated and destructed. (**A**) Depletion of PD-L1 increased E-cadherin transcription but had no effect on Snail transcription. MDA-MB-231 cells were treated with control or each of 2 distinct PD-L1 siRNAs for 48 hours. mRNA levels of E-cadherin and Snail in these cells were examined by real-time quantitative PCR. (**B**) Reexpression of PD-L1 suppressed E-cadherin transcription. MDA-MB-231 cells were transfected with mock or PD-L1–expressing construct for 24 hours, then transfected with control (siNC) or PD-L1 siRNA (siPD-L1–2) for another 48 hours. The mRNA levels of E-cadherin in each group were examined by RT-qPCR. (**A** and **B**) Data (*n* = 3 independent experiments) were normalized against the siNC group, plotted as mean ± SEM, and statistically analyzed using unpaired 2-tailed Student’s *t* test with the *P* value adjusted by Bonferroni’s method. *, *P* < 0.05; **, *P* < 0.01; ***, *P* < 0.001. (**C**) Inhibition of proteasome recovered the loss of Snail in PD-L1–depleted cells. Control (siNC) or PD-L1–depleted (siPD-L1–2) MDA-MB-231 cells were treated with 10 μM MG132 for 6 hours. Cells were then lysed and subjected to immunoblotting with indicated antibodies. (**D**–**F**) Snail ubiquitination was enhanced in PD-L1–depleted cells in a GSK3β-dependent manner. (**D**) Parental and PD-L1–null (KO-1) MDA-MB-231 cells were transfected with HA-tagged Snail and Myc-tagged ubiquitin for 48 hours. (**E**) MDA-MB-231 cells were transfected with HA-Snail and Myc-ubiquitin for 24 hours, then transfected with control (siNC) or PD-L1 (siPD-L1–2) siRNA for 48 hours. (**F**) Cells described in **E** were treated with DMSO or 10 μM SB216763 for 6 hours. (**D**–**F**) After being treated with MG132 (10 μM, 6 hours), cells were lysed with denatured IP buffer, then subjected to immunoprecipitation (IP) with indicated antibodies (HA antibody for **F**). The precipitates were analyzed by immunoblotting using indicated antibodies. Intensity of ubiquitinated Snail was quantified by ImageJ (NIH) and normalized against control. (**D**–**F**) Data (*n* = 3 independent experiments) were normalized against the parental (**D**), siNC (**E**), or siNC/DMSO (**F**) group; plotted as mean ± SEM; and statistically analyzed using unpaired 2-tailed Student’s *t* test. *, *P* < 0.05; **, *P* < 0.01. N.S., no significant difference.

This effect of PD-L1 is very likely conducted via regulating the EMT, in the context that the Snail family TFs and the EMT play important roles in stimulating cancer metastasis and progression not only by improving migration and invasiveness but also by conferring tumor cells with stem cell–like traits that enhance the ability of tumor cells to survive in foreign microenvironments, such as in circulation and distant organs (20, 24). To gain insights into the underlying mechanism by which PD-L1 regulates the EMT, we took a closer look at E-cadherin and Snail, 2 key EMT-related proteins that showed significant changes when PD-L1 expression was modified (Figure 1, A and B). Levels of EMT-TFs determine the status of the cell on the EMT spectrum from complete epithelial to complete mesenchymal by regulating the expression of epithelial proteins (such as E-cadherin, ref. 20) and mesenchymal proteins (such as fibronectin, ref. 24). Results from quantitative reverse transcription PCR (RT-PCR) analyses revealed an escalation of E-cadherin mRNAs not only in PD-L1–null cells but also in siRNA-treated PD-L1–knockdown cells (Figure 3A), supporting a causal relationship between E-cadherin and PD-L1 in MDA-MB-231 cells. Importantly, restored expression of PD-L1 diminished the increased transcription of E-cadherin in PD-L1–knockdown cells (Figure 3B). These results align well with the increase of E-cadherin proteins and decrease of Snail proteins when PD-L1 was depleted (Figure 1, A and B). Thus, we reason that PD-L1 inhibits E-cadherin transcription by upregulating Snail expression.

However, mRNA levels of Snail were not changed in PD-L1–knockdown cells (Figure 3A), although Snail transcription appeared impaired in the 2 PD-L1–null clones (Supplemental Figure 4A). This indicates that PD-L1 more likely plays a consistent role in regulating Snail at a posttranscriptional level than at the transcriptional level. As a critical TF promoting the EMT program and regulating cell survival/differentiation (20, 25, 26), Snail is under tight control in cells. The ubiquitination-dependent, proteasome-mediated destruction limits the protein level of Snail and makes Snail a short-lived protein (27). Interestingly, treatment with proteasome inhibitor MG132 strongly elevated the protein level of Snail in PD-L1–knockdown cells to a comparable level to that in parental cells (Figure 3C). Similar results were obtained in PD-L1–null cells (Supplemental Figure 4B), confirming that PD-L1 has more consistent influence on Snail stability than transcription. Because proteasome-mediated protein destruction depends on ubiquitination, we next determined Snail ubiquitination in parental and PD-L1–deficient cells. To eliminate the possibility that some of the ubiquitin signal accumulated via immunoprecipitation was not from ubiquitinated Snail but from other ubiquitinated, Snail-binding proteins, we employed the denatured immunoprecipitation in which the immunoprecipitation of Snail was performed using denatured cell lysates to limit the noncovalent binding of other proteins to Snail. Using this approach, we showed that exogenously expressed HA-tagged Snail was much more heavily ubiquitinated in PD-L1–null (Figure 3D) or PD-L1–knockdown (Figure 3E) cells. Moreover, endogenous Snail also exhibited higher level of ubiquitination in PD-L1–depleted cells than in parental cells (Supplemental Figure 4C). Our data suggest an intriguing possibility that the expression of PD-L1 in TNBC cells mediates a tumor-intrinsic signaling that inhibits Snail ubiquitination, thus promoting the EMT program.

### PD-L1 inhibits GSK3β activity by activating p38-MAPK.

**Figure 4:**
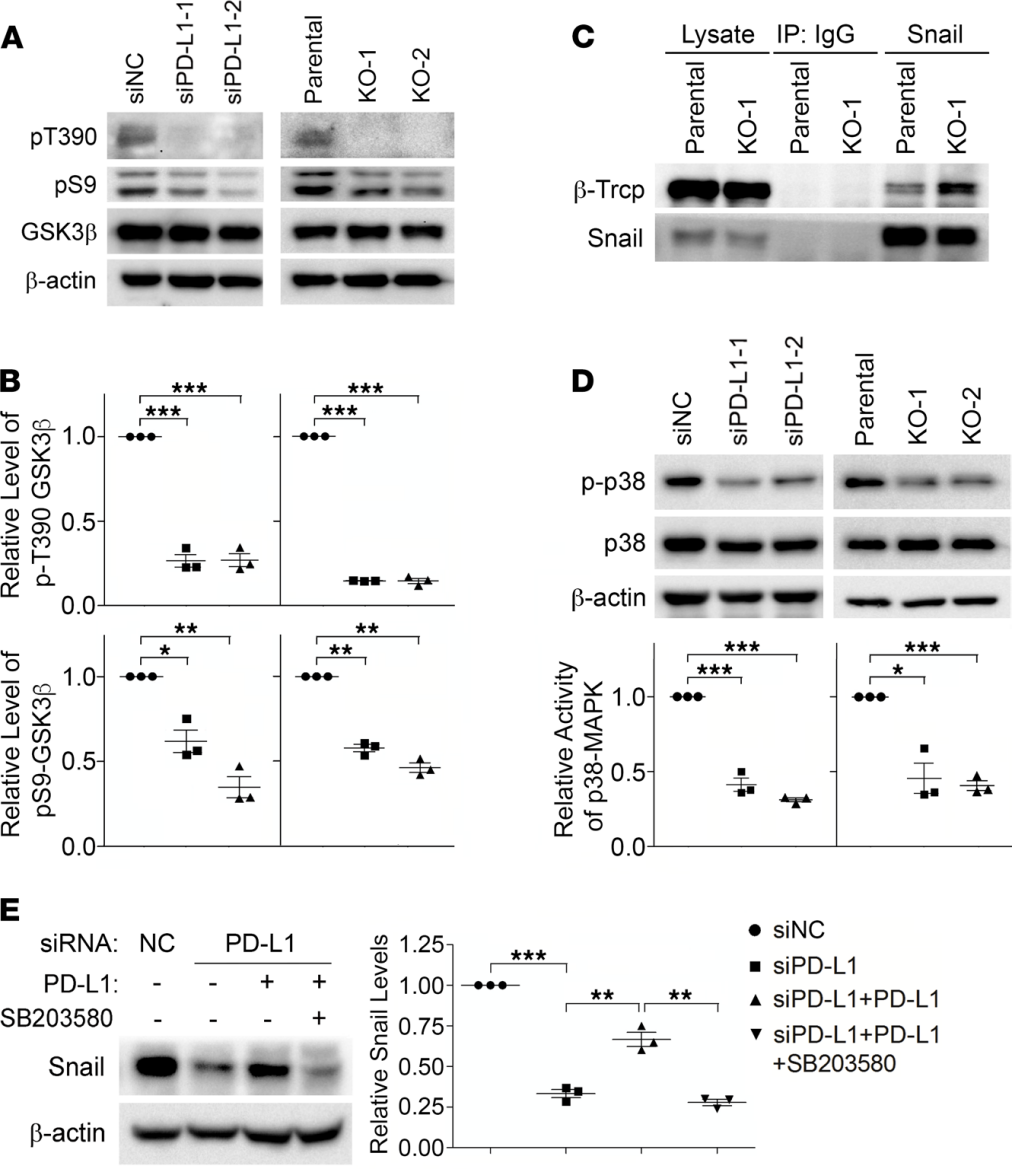
PD-L1 prevents Snail ubiquitination via p38-MAPK–mediated inhibition of GSK3β. (**A** and **B**) The inhibitory phosphorylations of GSK3β (pT390 and pS9) were decreased in PD-L1–deficient cells. (**A**) Control, PD-L1–knockdown, and PD-L1–knockout MDA-MB-231 cells were analyzed by immunoblotting using indicated antibodies. (**B**) The intensity of pT390-GSK3β and pS9-GSK3β were measured and normalized against total GSK3β in each group. Results was then normalized against the control group. (**C**) Snail exhibited stronger association with β-Trcp in PD-L1–deficient MDA-MB-231 cells. Endogenous Snail was immunoprecipitated from parental or KO-1 MDA-MB-231 cells. β-Trcp associated with Snail was determined by immunoblotting. (**D**) PD-L1–deficient cells exhibited significantly less p38-MAPK activity. The activating phosphorylation of p38-MAPK (p-p38) was determined by immunoblotting. The relative activity of p38-MAPK was represented by the ratio of p-p38 to total p38. (**E**) Selective inhibition of p38-MAPK suppressed the PD-L1–induced expression of Snail. MDA-MB-231 cells stably expressing PD-L1 were established by lentivirus-mediated infection. These cells were pretreated with SB203580 (10 μM) for 2 hours before transfection with siNC or siPD-L1 for 48 hours with SB203580, then subjected to immunoblotting analysis using indicated antibodies. After normalizing against β-actin levels, the expression level of Snail in each group was quantified. (**B** and **D**) Results (*n* = 3 3 independent experiments) were plotted as mean ± SEM and statistically analyzed using unpaired 2-tailed Student’s *t* test with the *P* value adjusted by Bonferroni’s method. *, *P* < 0.05; **, *P* < 0.01; ***, *P* < 0.001. (**E**) Results (*n* = 3 independent experiments) were plotted as mean ± SEM and statistically analyzed using 1-way ANOVA with the *P* value adjusted by Tukey’s honestly significant differences (HSD) using R function “aov” and “TukeyHSD” from package “stats” in R version 3.6.3. **, *P* < 0.01; ***, *P* < 0.001.

Snail ubiquitination is catalyzed by E3 ubiquitin ligase complexes, mainly the Skp1-Cullin-F-box (SCF) protein complexes (27). Phosphorylation of Snail enhances its binding to the substrate-recruiting F-box protein of these SCF E3 ligases and facilitates the subsequent ubiquitination (27). GSK3β-mediated phosphorylation of Snail enhances the binding of Snail with F-box protein β-transducin repeat-containing protein (β-Trcp) and Snail ubiquitination by SCF^β-Trcp^ (28). Indeed, levels of ubiquitinated Snail (Figure 3F), as well as the global ubiquitination (Supplemental Figure 4D), were comparable in parental and PD-L1–depleted MDA-MB-231 cells after treatment with GSK3β-specific inhibitor, suggesting that GSK3β is required for the increase of Snail ubiquitination caused by PD-L1 depletion. Additionally, we found that in PD-L1–null and PD-L1–knockdown cells, GSK3β phosphorylation at threonine 390 (T390) was almost completely suppressed compared with that in control cells (Figure 4, A and B). Moreover, the phosphorylation of serine 9 (S9) in GSK3β was also slightly weakened when PD-L1 expression was suppressed (Figure 4, A and B). Because phosphorylations at T390 and S9 are both inhibitory to GSK3β activity (29), our results suggest that loss of PD-L1 enhances GSK3β activity, which subsequently promotes the phosphorylation and ubiquitination of Snail. Although GSK3β-mediated phosphorylation on Snail cannot be directly determined due to the lack of specific antibodies, we indeed found that the association of β-Trcp with Snail was significantly increased when PD-L1 was depleted (Figure 4C). Protein kinase D (PKD) (30) was also reported to facilitate Snail ubiquitination by phosphorylating Snail at S11 and enhancing its interaction with another F-box protein, FBXO11. However, we did not see obvious changes of PKD activity in PD-L1–depleted cells by determining the phosphorylation at T95 of PKDs (Supplemental Figure 5A); and correspondingly, no change of Snail phosphorylation at S11 was observed in parental and PD-L1–deficient cells using specific antibody (30) (Supplemental Figure 5B). These data suggest that PD-L1 in TNBC cells suppresses the interaction of Snail with the E3 ubiquitin ligase SCF^β-Trcp^ by inhibiting GSK3β activity.

GSK3β phosphorylation at T390 is mediated by p38-MAPK (31), whereas S9 can be phosphorylated by the PI3K/AKT pathway (32) as well as by other kinases, including the ERK-induced activation of p90 ribosomal protein S6 kinase (33) and p70 ribosomal protein S6 kinase (p70S6K) (34–36). To understand how GSK3β is regulated by PD-L1, we compared the activity of these kinases in normal or PD-L1–depleted MDA-MB-231 cells. Compared with parental cells, ERK1/2 activation in PD-L1–knockdown or PD-L1–null cells was unchanged, and AKT activity in PD-L1–deficient cells was slightly increased (Supplemental Figure 5C). The change of p70S6K activity was inconsistent in PD-L1–knockdown cells and PD-L1–knockout cells (Supplemental Figure 5D), indicating that p70S6K might not be a constant contributor to PD-L1–dependent inhibitory phosphorylation of GSK3β. On the other hand, we observed a significant, consistent decrease of p38-MAPK activity in both PD-L1–knockdown and PD-L1–null cells compared with parental cells (Figure 4D and Supplemental Figure 3E), suggesting that PD-L1 expression promotes GSK3β phosphorylation at T390 by regulating the activity of p38-MAPK. In Figure 1B, we showed that reexpression of PD-L1 recovered the suppressed Snail expression in PD-L1–depleted cells. Notably, this PD-L1–induced upregulation of Snail could be completely blocked by p38-MAPK inhibitor (Figure 4E). Together, our data strongly suggest that PD-L1 when upregulated in TNBC cells can inhibit GSK3β by activating p38-MAPK. This prevents Snail from being phosphorylated by GSK3β and caught by SCF^β-Trcp^ for ubiquitination and destruction. We reason that this PD-L1–dependent protection of Snail leads to Snail accumulation and then promotes the EMT and aggressiveness of PD-L1–expressing TNBC cells. Consistent with this conclusion, we observed that levels of Snail and activated p38 are positively associated with PD-L1 levels in TNBC patient tissues (Supplemental Figure 5E).

### PD-L1 directly interacts with and inhibits PTP1B.

**Figure 5:**
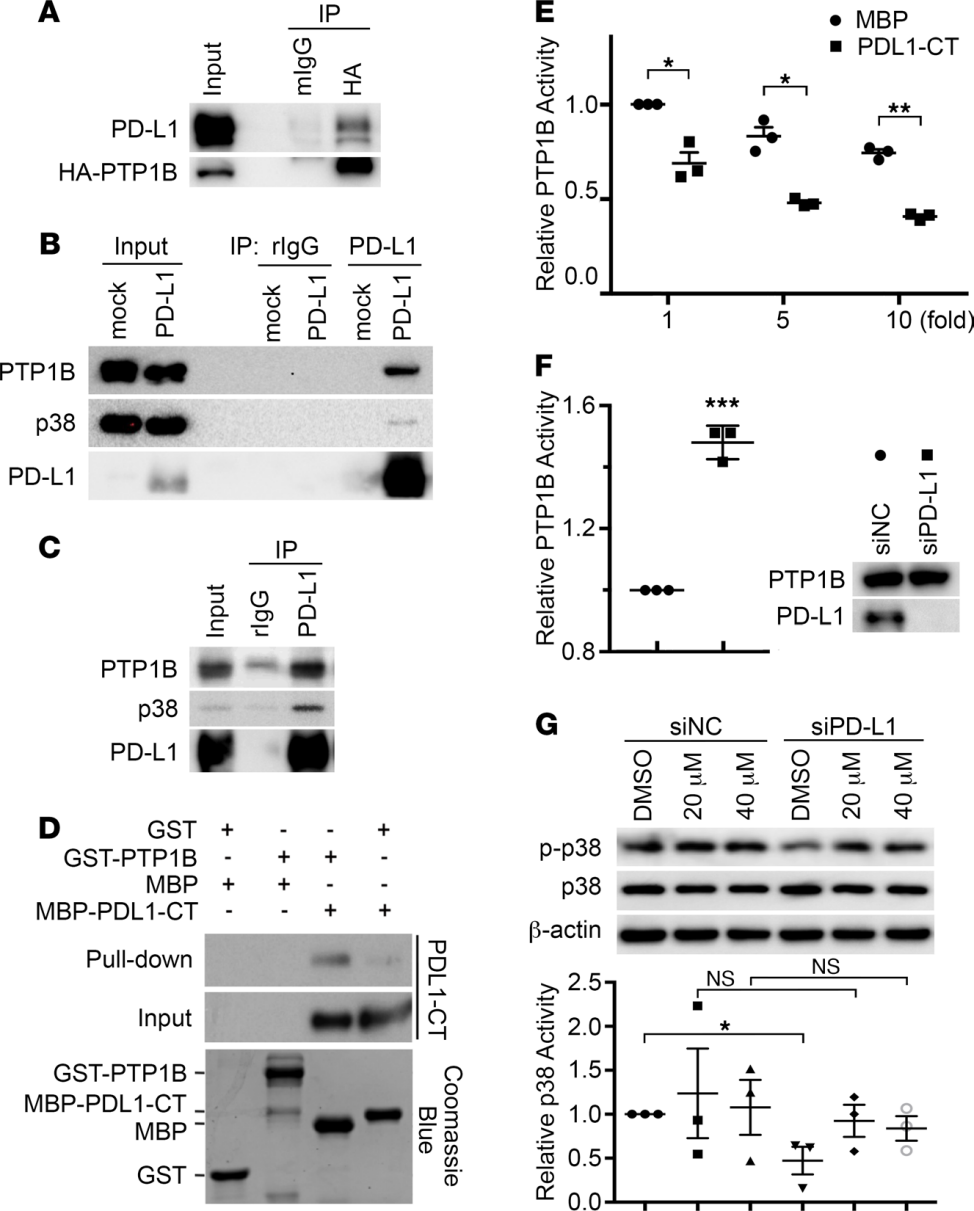
PD-L1 directly interacts with PTP1B and inhibits its phosphatase activity. (**A**) PD-L1 and HA-tagged PTP1B associate with each other. HEK293T cells were transfected with PD-L1 and HA-tagged PTP1B for 48 hours and then subjected to immunoprecipitation followed by immunoblotting. (**B**) Ectopically expressed PD-L1 could pull down endogenous PTP1B and p38-MAPK. PD-L1 overexpressed in HEK293T was immunoprecipitated by PD-L1 antibody and the associated PTP1B were visualized by immunoblotting. (**C**) Endogenous PD-L1, PTP1B, and p38-MAPK form a protein complex in MDA-MB-231 cells. (**D**) The cytoplasmic domain of PD-L1 directly interacts with PTP1B. GST-tagged PTP1B (GST-PTP1B) and MBP-tagged PD-L1 cytoplasmic domain (MBP-PDL1-CT) were purified from *E*. *coli*. Purified proteins were analyzed by SDS-PAGE followed by Coomassie blue staining (lower panel). GST pull-down assays were performed using 0.5 g of each indicated protein. (**E** and **F**) PD-L1 inhibited the phosphatase activity of PTP1B. In vitro phosphatase assay was performed using 120 ng purified GST-PTP1B with indicated amount of MBP or MBP-PDL1-CT (**E**) or using endogenous PTP1B immunoprecipitated by anti-PTP1B antibody from indicated cell lysates (**F**). PTP1B activity in each sample was normalized against PTP1B with equal amount of MBP (**E**) or cells treated with control siRNA (siNC, **F**, immunoprecipitants pulled down from control cells by normal mouse IgG were used as negative controls of the phosphatase activity assay). (**G**) Inhibition of PTP1B recovered p38-MAPK activity in PD-L1–deficient cells. MDA-MB-231 cells were transfected with control (siNC) or PD-L1 (siPD-L1–1) siRNAs along with PTP1B inhibitor (20 or 40 μM) for 48 hours, then lysed for immunoblotting using indicated antibodies. The relative intensity of phosphorylated p38-MAPK (p-p38) in each sample was determined by GraphPad and normalized against the total p38-MAPK. (**E**–**G**) Results (*n* = 3 independent experiments) were plotted as mean ± SEM and comparisons between indicated groups were statistically analyzed using unpaired 2-tailed Student’s *t* test. *, *P* < 0.05; **, *P* < 0.01; ***, *P* < 0.001.

PD-L1 is a single transmembrane protein with a short cytoplasmic tail. To investigate how PD-L1 activates p38-MAPK, we employed a proximity-dependent biotin identification (BioID) approach to label the proteins interacting with PD-L1. For this purpose, the carboxyl end of PD-L1 was fused to a mutated bacterial biotin ligase BirA* with a labeling radius of approximately 10 nm (37), and this fusion protein was stably expressed in PD-L1–null MDA-MB-231 cells. After being labeled with biotin, the biotinylated proteins were pulled down from cells by streptavidin-conjugated beads and then analyzed by mass spectrometry. Compared with cells expressing BirA* alone, we identified proteins that potentially associate with PD-L1, and one of them was the protein tyrosine phosphatase PTP1B (also called PTPN1), which is highly correlated with tumorigenesis and progression of various cancers (38). By coexpressing exogenous PTP1B and PD-L1 in human embryonic kidney 293T (HEK293T) cells, we confirmed that PTP1B and PD-L1 associated with each other (Figure 5A). Further, overexpressed PD-L1 and endogenous PTP1B formed a protein complex, within which endogenous p38-MAPK was also detected (Figure 5B). More importantly, endogenous PD-L1 successfully pulled down endogenous PTP1B as well as p38-MAPK in MDA-MB-231 cells (Figure 5C), supporting the presence of p38-MAPK in the PD-L1–PTP1B complex. To determine whether PD-L1 directly binds to PTP1B, we constructed GST-tagged PTP1B (GST-PTP1B) and MBP-tagged PD-L1 cytoplasmic domain (MBP-PDL1-CT). These proteins were expressed and purified from *E*. *coli* (Figure 5D), then used for in vitro protein pull-down assay. Results shown in Figure 5D confirmed the direct interaction of PTP1B with the cytoplasmic domain of PD-L1. Furthermore, truncated PTP1B proteins lacking the C-terminal ER-targeting domain and the intact proline-rich domain cannot interact with MBP-PDL1-CT (Supplemental Figure 6, A and B), suggesting that one or both of these domains mediates the interaction with PD-L1.

PTP1B could inhibit p38-MAPK by dephosphorylating p38 at tyrosine 182 (T182) (39). T182 is one of the 2 sites that when phosphorylated activate p38-MAPK (40, 41), and this phosphorylation can be recognized by the phosphorylated p38 antibody used in our experiments. The direct interaction of PD-L1 cytoplasmic domain with PTP1B raises an intriguing possibility that PTP1B might play a role in the PD-L1–dependent regulation of p38-MAPK. Indeed, results from the in vitro PTP1B phosphatase assay showed that MBP-PDL1-CT, but not MBP alone, inhibited PTP1B activity in a dose-dependent manner (Figure 5E). This suggests that PD-L1 by binding to PTP1B can inhibit PTP1B-mediated dephosphorylation and inactivation of p38-MAPK. Although the PD-L1–associated PTP1B was a small portion of total PTP1B in resting MDA-MB-231 cells (Supplemental Figure 6C), we indeed observed a moderate, yet significant, increase of PTP1B activity when PD-L1 was depleted (Figure 5F). Moreover, PTP1B inhibitor recovered the decreased p38-MAPK phosphorylation in PD-L1–depleted cells to a level comparable to that in parental cells (Figure 5G), further confirming the participation of PTP1B in the PD-L1–dependent activation of p38-MAPK.

### The tumor-intrinsic signaling of PD-L1 can be activated by extracellular stimuli.

**Figure 6:**
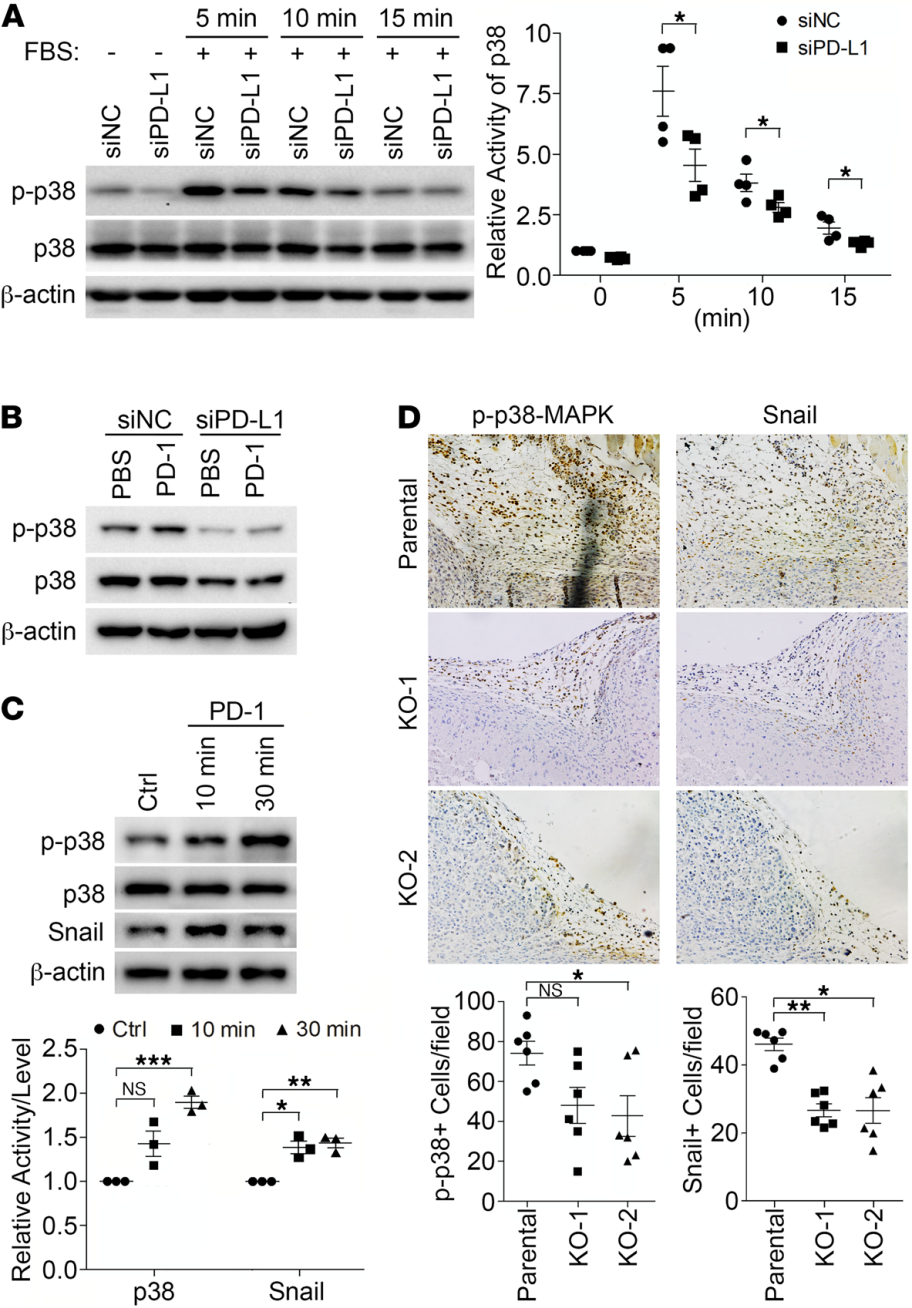
PD-L1 mediates a tumor-intrinsic signaling that can be activated by PD-1. (**A**–**C**) Cells were subjected to immunoblotting with indicated antibodies. (**A**) PD-L1 is necessary for the activation of p38-MAPK by extracellular stimuli. MDA-MB-231 cells were transfected with control or PD-L1 siRNAs for 48 hours, serum-starved overnight, and then treated with FBS (10%) for indicated amount of time. (**B**) PD-1 activates p38-MAPK in a PD-L1–dependent manner. Control or PD-L1–depleted MDA-MB-231 cells were treated with PBS or PD-1 (0.5 μg/mL) for 15 minutes. (**C**) PD-1 treatment increased the protein levels of Snail in MDA-MB-231 cells. Cells were treated with PBS or PD-1 for 10 or 30 minutes. (**A** and **C**) The relative activity of p38 and protein level of Snail were quantified. Data (*n* = 3 independent experiments) were plotted as mean ± SEM and statistically analyzed using unpaired 2-tailed Student’s *t* test with (**C**) or without (**A**) the *P* value adjusted by Bonferroni’s method. N.S., no significant difference; *, *P* < 0.05; **, *P* < 0.01; ***, *P* < 0.001. (**D**) PD-L1 is required for p38 activation and Snail expression in vivo. MDA-MB-231 tumors grown in NOD/SCID mice as described in Figure 2 were collected and processed for immunohistochemistry staining to determine p38 activity and Snail expression in tandem tissue slides. Power of eyepiece: 10×; power of objective: 20×. Positive cells were counted in >10 fields/slide/mice, averaged, and plotted as mean ± SEM. Data (*n* = 6 mice/group) were statistically analyzed using unpaired 2-tailed Student’s *t* test with the *P* value adjusted by Bonferroni’s method. N.S., no significant difference; *, *P* < 0.05; **, *P* < 0.01.

Our current findings raise an intriguing possibility that the single transmembrane PD-L1 can function as a receptor to mediate tumor-intrinsic signaling. The PD-L1–mediated signaling could be activated by conformational changes in PD-L1, which may be triggered by ligands binding to its extracellular domain. Another possibility is that PD-L1 may function as a coreceptor. In MDA-MB-231 cells, we did not detect the expression of CD80 or PD-1 (Supplemental Figure 1D), 2 natural PD-L1 binding partners that may induce PD-L1 conformational changes. To determine how PD-L1 influences intracellular signaling pathways in cultured cells, we examined the p38 activation induced by FBS in parental and PD-L1–deficient MDA-MB-231 cells. As shown in Figure 6A, the FBS-induced p38-MAPK activity in PD-L1–deficient cells was significantly weaker than that in control cells. These results suggest that PD-L1 not only boosts p38-MAPK activity to a higher level in resting cells but also synergizes with other extracellular stimuli to further enhance p38-MAPK activation. The hyperactivated p38-MAPK in PD-L1–overexpressing cells, which could result from PD-L1–dependent PTP1B inhibition, subsequently constrains GSK3β activity and protects Snail from being destructed.

To test if the PD-L1 tumor-intrinsic pathway can be activated by its binding partner, we treated MDA-MB-231 cells with purified recombinant PD-1 extracellular domain, then determined p38-MAPK activity. Our results showed that PD-1 ectodomain activated p38-MAPK in parental MDA-MB-231 cells; however, PD-L1 depletion inhibited p38 activation induced by PD-1 (Figure 6B). Additionally, PD-1 treatment also caused an increase of Snail in parental MDA-MB-231 cells (Figure 6C). Together, these results suggest that PD-1 binding to PD-L1 can activate the PD-L1 intrinsic tumor-promoting pathway in TNBC cells. Thus, PD-L1 and PD-1 may be ligand and receptor to each other mutually. To determine if this is the case in vivo, we analyzed the primary tumors from our orthotopic xenograft studies described above. Immunohistochemistry analysis showed strong p38-MAPK phosphorylation in control tumors, especially at the peripheral area that represents the interface of tumor mass and the host tissue; however, PD-L1–null tumors showed notably less phosphorylated p38-MAPK, with the KO-2 group showing significant difference from control tumors (Figure 6D). When we examined Snail expression in the same tumor areas, the numbers of Snail-positive cells in PD-L1–null tumors were significantly decreased compared with those in control tumors (Figure 6D). These results suggest that the p38-MAPK activity and Snail expression level in parental MDA-MB-231 tumors are PD-L1 dependent. Since the activated p38-MAPK signal was only observed at the peripheral region of tumors, we reason that the tumor-associated microenvironment may activate PD-L1 on tumor cells. Although the immunodeficient NOD/SCID mice do not have B and T cells, macrophages accumulated in tissues surrounding tumors where PD-1 expression was positive (Supplemental Figure 7A). Moreover, we showed that mouse PD-1 can bind to human PD-L1 (Supplemental Figure 7B), and this binding can be blocked by our anti–mouse PD-1 antibody (αmPD-1, Supplemental Figure 7C), as suggested previously (42). Interestingly, αmPD-1 treatment suppressed the progression of parental MDA-MB-231 tumors in NOD/SCID mice but had no effect on PD-L1–deficient MDA-MB-231 tumors (Supplemental Figure 7D). In the context that MDA-MB-231 cells do not express PD-1 (Supplemental Figure 1D), these data suggest that the binding of PD-1 in microenvironment to PD-L1 in tumor cells favors tumor progression. Thus, we reason that host cells in the tumor-associated microenvironment, such as macrophages, produce PD-1 to activate the PD-L1 pathway in tumor cells. Moreover, p38-MAPK activity in tumor cells, which can be stimulated by growth factors and cytokines produced by tumor-associated host cells, would be sustained at higher level for longer time in PD-L1–expressing tumor cells. Together, these effects could protect Snail and promote the EMT and tumor aggressiveness.

### PD-L1 antibodies inhibit TNBC progression independent of antitumor immunity.

**Figure 7:**
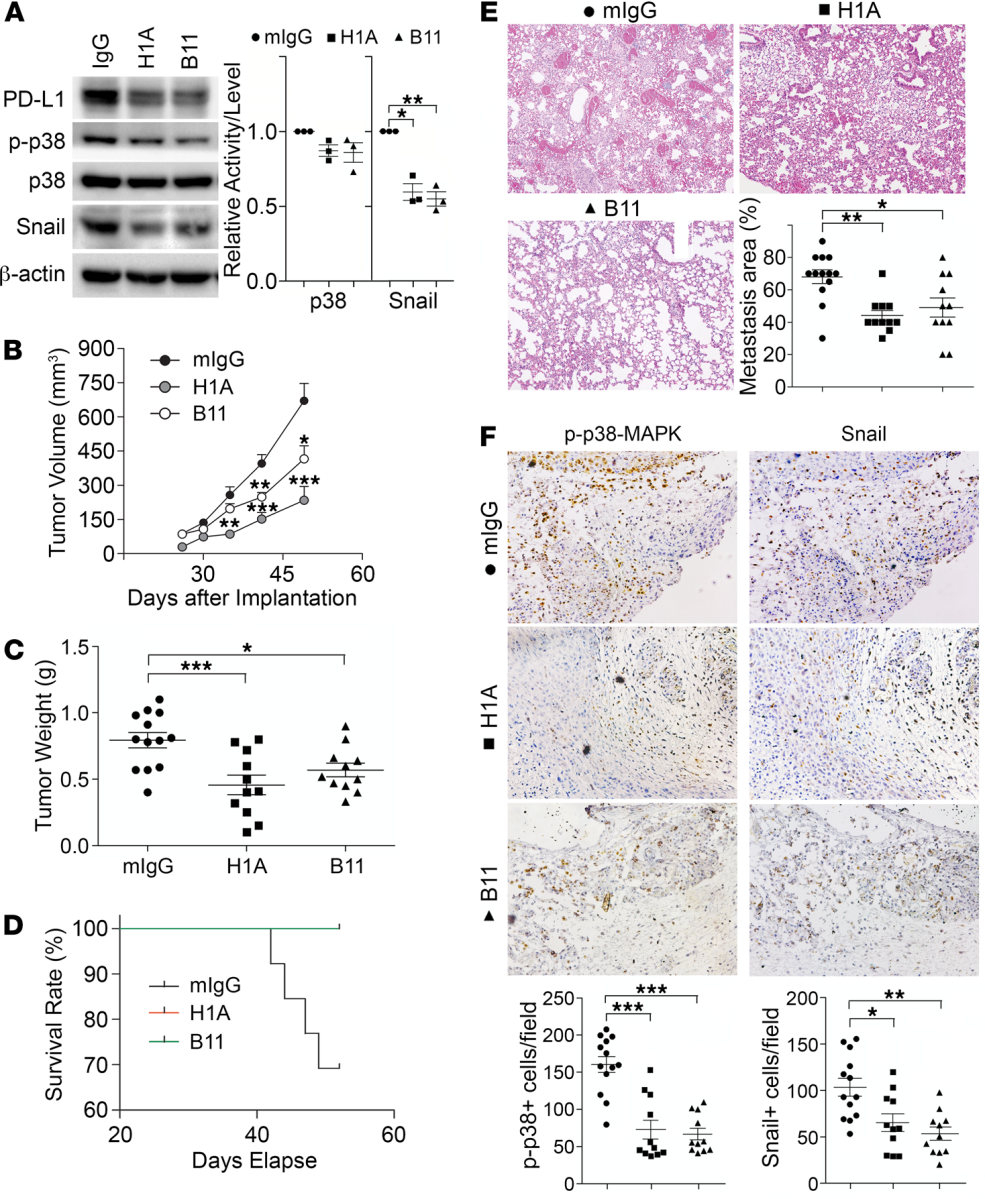
PD-L1 antibodies diminish PD-L1 tumor-intrinsic signaling and inhibit TNBC progression independent of antitumor immunity. (**A**) PD-L1 antibodies suppressed PD-L1 expression and signaling. MDA-MB-231 cells were treated with control IgG, H1A, or B11 for 48 hours before being subjected to immunoblotting. Relative p38 activity was calculated as the ratio of phosphorylated p38 and total p38, then normalized against the control group (IgG). Data were plotted as mean ± SEM and statistically analyzed using unpaired 2-tailed Student’s *t* test with the *P* value adjusted by Bonferroni’s method (*n* = 3 independent experiments). *, *P* < 0.05; **, *P* < 0.01. (**B**–**F**) NOD/SCID mice receiving 2 × 10^6^ MDA-MB-231 cells in mammary fat pad were separated into 3 treatment groups (IgG, H1A, or B11). Starting from day 4 after inoculation, 200 μg antibodies were administrated intraperitoneally every 3 days until termination. (**B** and **C**) Tumor was measured weekly (**B**) and weighed at the endpoint (**C**). (**D**) Survival (Kaplan-Meier) curve was summarized in each treatment group. (**E**) Micrometastatic lesions in lung tissues from each treatment group were visualized by H&E staining and quantified. Power of eyepiece: 10×; power of objective: 10×. (**F**) Treatment of PD-L1 antibodies inhibited PD-L1 signaling. The number of phosphorylated p38-positive or Snail-positive cells in tumor tissues was determined by immunohistochemistry staining in tandem tissue slides prepared from tumors treated with control or PD-L1 antibodies. Power of eyepiece: 10×; power of objective: 20×. (**E** and **F**) Values from more than 10 fields in slide from each mouse were quantified and averaged. (**B**–**F**) Data for each group were plotted as mean ± SEM, and comparisons with the mouse IgG group were statistically analyzed using 1-way ANOVA analysis with Dunnett’s test (*n* = 11–13 mice/group). *, *P* < 0.05; **, *P* < 0.01; ***, *P* < 0.001.

That PD-1 can activate PD-L1 in tumor cells raises a possibility that the binding of antibodies to its extracellular domain may also affect the PD-L1–mediated tumor-intrinsic signaling. Subsequently, the tumor-promoting function of PD-L1 could be interrupted, which may suppress tumor progression. To test this, we employed 2 homemade monoclonal antibodies of PD-L1, named H1A and B11. According to epitope analysis, H1A and B11 recognize distinct regions in the extracellular domain of human PD-L1. Our most recent work (43) showed that H1A binding promotes the degradation of PD-L1 by eliminating the interaction of PD-L1 with its protective binding partner, CMTM6 (44, 45). As shown in Figure 7A, treatment of H1A and B11 both led to a significant reduction of PD-L1 protein levels, consistent with our previous report (43). The p38-MAPK activity was slightly decreased in these cells, which is likely because the level of remaining PD-L1 in these cells was much higher than in PD-L1–deficient cells. Nevertheless, a robust decrease of Snail was observed in H1A- and B11-treated cells (Figure 7A). These results suggest that H1A and B11 attenuated the tumor-intrinsic function of PD-L1, likely by promoting PD-L1 degradation, which may then inhibit tumor progression independent of immune response. To investigate this possibility, we treated the immunodeficient NOD/SCID mice carrying orthotopically transplanted MDA-MB-231 tumors with H1A and B11.

To maximize the blockade potential of PD-L1 antibodies, we pretreated parental MDA-MB-231 cells with 1 mg control IgG, H1A, or B11 for 30 minutes. These cells were subcutaneously inoculated into the mammary fat pad of NOD/SCID mice. Three days later, animals in each group were treated with control IgG, H1A, or B11, respectively, via intraperitoneal injection every 3 days until the planned endpoint (day 52 after inoculation). As shown in Figure 7B, tumors treated with both H1A and B11 grew notably slower than tumors treated with control IgG, with H1A achieving stronger inhibition of tumor growth. By day 49, 30% of animals treated with control IgG had met the terminating body conditions; however, mice treated with H1A or B11 all appeared normal. We terminated the study at day 52 and analyzed the primary tumors and lung metastasis in all animals. The weight of primary tumors in mice treated with control IgG was significantly higher than tumors treated with PD-L1 antibodies (Figure 7C). Correspondingly, all mice treated by H1A or B11 survived until the endpoint, whereas 4 out of 13 mice treated by control IgG met the terminating body condition (Figure 7D). Because no metastatic nodules on the lung surface were observed in all animals, including mice in the control IgG group in this particular study, we determined the micrometastatic lesions using histological analyses. As shown in Figure 7E, animals treated with both PD-L1 antibodies developed significantly fewer metastatic lesions in lung than those treated with control IgG, which is consistent with the much better survival curves of these treatment groups.

Because NOD/SCID mice lack B and T cells, the tumor-suppressing effect of H1A and B11 is independent of the antitumor immune response. Compared with tumors treated by control IgG, tumors treated by H1A or B11 showed markedly less p38-MAPK activity (Figure 7F, left) and Snail expression (Figure 7F, right). Yet, the expression of PD-1 or the number of macrophages in the tumor-associated microenvironment remained comparable in mice treated by empty vehicle or H1A/B11 (data not shown), indicating that the intrinsic tumor-promoting function of PD-L1 was impaired by H1A or B11. These data suggest that PD-L1–expressing tumors likely require the tumor-intrinsic signaling of PD-L1 to develop aggressive behaviors, such as metastasis. Together, our results indicate that PD-L1 antibodies that trigger PD-L1 internalization and degradation would fit the need to abolish both the intrinsic and extrinsic functions of PD-L1 in TNBC cells.

### Targeting both tumor-intrinsic and -extrinsic functions of PD-L1 synergistically suppress TNBC.

When characterizing our homemade anti–human PD-L1 antibodies, we found that H1A and B11 could not block the binding of PD-1 to PD-L1 (Supplemental Figure 8A), although both of them bound to PD-L1 with high affinity similar to atezolizumab, the FDA-approved PD-1–blocking PD-L1 antibody (Supplemental Figure 8B). This makes it possible to test whether targeting the tumor-intrinsic function of PD-L1 can provide additional benefit for TNBC patients when combined with the PD-1 antibody that inhibits PD-L1 binding. For this purpose, we employed E0771 cells, which are syngeneic TNBC cells from C57BL/6 mice and express mouse PD-L1. To utilize the human PD-L1 antibody H1A that does not block PD-1 binding, we first humanized E0771 cells (E0771-hPDL1) by knocking out the endogenous mouse PD-L1 using CRISPR/Cas9 (Supplemental Figure 8C) and then stably expressing human PD-L1 in them (Supplemental Figure 8D). These E0771-hPDL1 cells were injected into the mammary fat pad of female C57BL/6 mice to create an immunocompetent, syngeneic TNBC mouse model. On day 11 after inoculation, when the tumor volume reached approximately 40 mm^3^, we divided mice into 4 groups and treated them with 200 μg control IgG, αmPD-1 that suppresses tumor growth by blocking the binding between mouse PD-1 and human PD-L1 (Supplemental Figure 7, B–D), H1A, or 1:1 mixed αmPD-1 and H1A, respectively. Five treatments were administrated with 3-day interval between each. Then, we monitored the tumor growth and body condition score of animals and terminated the ones with body condition score reaching 1 for tissue collection (primary tumor and lung). On day 74, when at least half of animals in each experimental group had been terminated, we ended the study and then collected primary tumors and lungs from the remaining mice.

**Figure 8:**
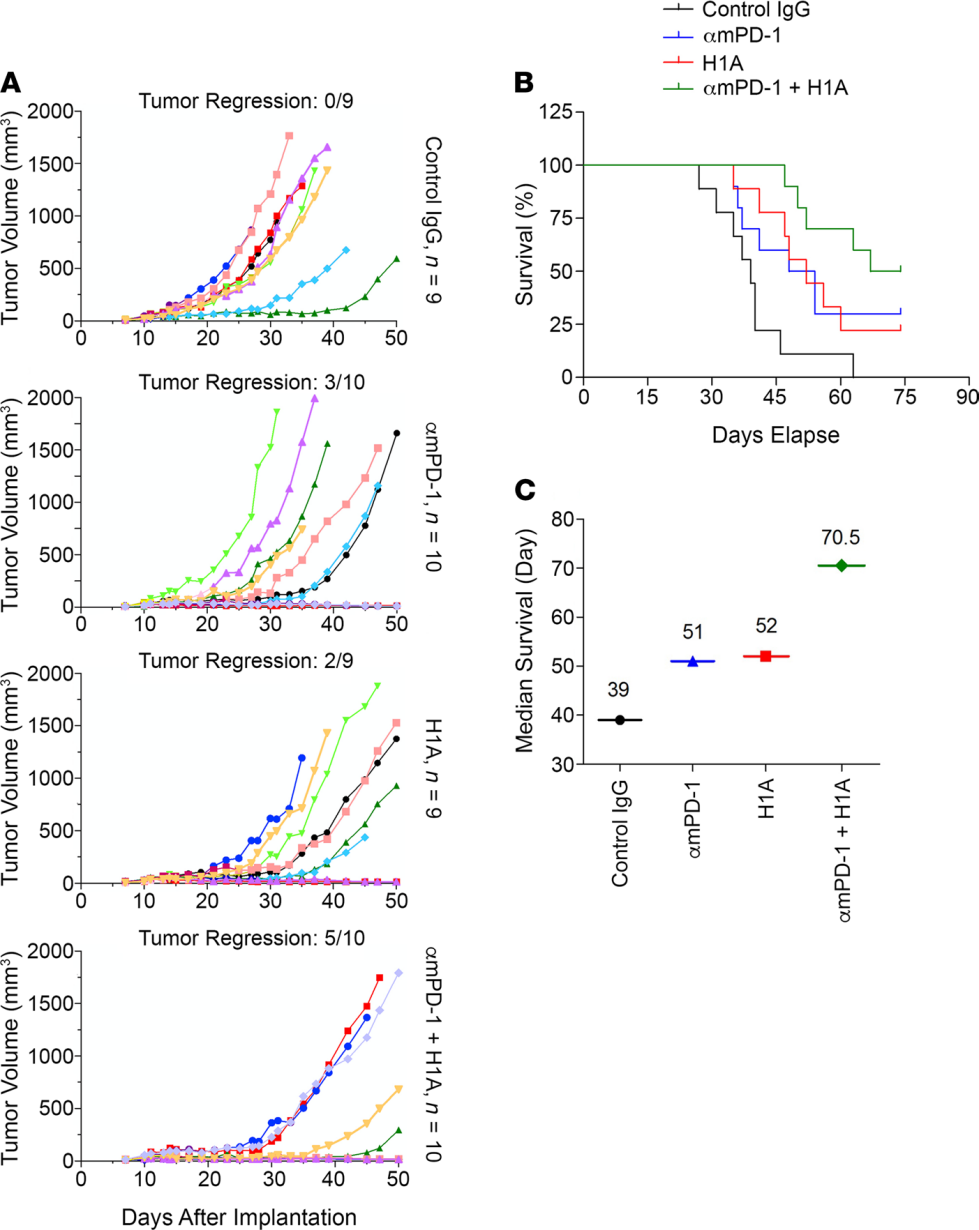
Targeting the tumor-intrinsic function of PD-L1 synergistically suppresses TNBC progression when combined with immune checkpoint blockade reagents. Humanized E0771 cells (1 × 10^6^), in which endogenous mouse PD-L1 was knocked out and human PD-L1 was overexpressed, were injected into the mammary fat pad of female C57BL/6 mice. On day 11 after inoculation, when the solid tumor could be touched, mice were randomly separated into 4 groups, which were treated with 200 μg control IgG, hamster anti–mouse PD-1 antibody (αmPD-1), mouse anti–human PD-L1 antibody H1A, or 100 μg αmPD-1 plus 100 μg H1A, respectively. Antibodies were injected intraperitoneally once every 3 days for 5 injections in total. (**A**) Combined treatment of αmPD-1 and H1A exhibited strong synergistic effect on suppressing tumor growth. Tumor volume was measured with calipers weekly until day 50 after inoculation and calculated as V = 0.5 × LW^2^. Tumor regression was shown as ratio of number of animals showing tumor regression and total animal number in each treatment group. (**B**) Combined treatment of αmPD-1 and H1A synergistically increased the survival of mice carrying E0771 tumor. Animals were monitored until day 74 after inoculation, when at least half of mice in each experimental group met the terminating body condition. (**C**) The median survival days of mice in each treatment group were calculated and plotted, which clearly showed that combined treatment of αmPD-1 and H1A elongated the survival time of animals carrying E0771 tumor.

As summarized in Figure 8A, the growth of E0771 tumors was considerably slower in αmPD-1–treated or H1A-treated groups, with 3 out of 10 and 2 out of 9 mice showing tumor regression, respectively. Significantly, the combined treatment of αmPD-1 and H1A exhibited further enhanced tumor suppression effect and achieved tumor regression in 5 out of 10 mice (Figure 8A). When comparing the average increase of tumor size in each group, the αmPD-1/H1A group also exhibited slower growth than the αmPD-1 or H1A group (Supplemental Figure 8E). All 3 treatments suppressed lung metastasis, with the αmPD-1/H1A combined treatment showing slightly stronger effect (Supplemental Figure 8F). Notably, mice treated with αmPD-1 plus H1A accomplished the best survival curve among all treatment groups (Figure 8B). On day 74 when the study was completed, all mice in the control IgG group had died, whereas the surviving ones in the αmPD-1 group, H1A group, and αmPD-1/H1A group were all tumor-regressed or tumor-free. The median survival was 70.5 days for αmPD-1/H1A combined group, which was considerably longer than that for the control IgG group (39 days), αmPD-1 group (51 days), or H1A group (52 days) (Figure 8C). Thus, the combined treatment of αmPD-1 and H1A achieved a significantly improved outcome compared with each single agent. In the context that H1A does not interrupt PD-1 binding to PD-L1 (Supplemental Figure 8A), these results clearly support an exciting conclusion that targeting the tumor-intrinsic function of PD-L1 could provide extra benefits when combined with immune checkpoint blockade reagents.

## Discussion

A tumor-intrinsic function of PD-L1 in tumor metastasis was defined in this study. We reported here that the intracellular domain of PD-L1 preserves p38-MAPK activity by inhibiting PTP1B and subsequently GSK3β. As GSK3β-mediated phosphorylation prevents Snail from being ubiquitinated and degraded, PD-L1 facilitates TNBC metastasis via the p38-MAPK/Snail pathway that promotes the EMT of TNBC cells. Also, we found that PD-L1 antibodies (H1A and B11) that cannot block PD-1 binding but induce PD-L1 degradation can phenocopy PD-L1 deficiency to make TNBC less aggressive in growth and metastasis in vivo. Importantly, we showed that H1A synergistically suppressed TNBC progression when combined with PD-1 blockade antibody in immunocompetent mice. Thus, our study reveals an immune-independent way of PD-L1 to facilitate TNBC progression and supports a new therapeutic strategy for TNBC treatment.

A bidirectional crosstalk between the EMT status and the PD-L1 expression has been observed in multiple types of cancer, including Claudin-low TNBC patients (9, 17, 46). It was shown that the EMT may drive PD-L1 expression via ZEB1-dependent downregulation of micro RNA-200 (47). Yet, the molecular mechanism underlying the PD-L1–mediated EMT remains vague. In current study, we defined a potentially novel physical interaction between the cytoplasmic domain of PD-L1 and PTP1B, which inhibits PTP1B. Our results demonstrated a PTP1B/p38-MAPK/GSK3β/Snail signaling axis that connects PD-L1 to the EMT status and aggressiveness of TNBC tumors. The role of PTP1B in cancer appears highly context dependent (48, 49). PTP1B expression was shown necessary for the ErbB2-induced mammary tumorigenesis (50); however, in p53-null mice, loss of PTP1B accelerates lymphomagenesis (51). In BC patients, loss of functional p53 is most prevalent with TN/basal-like BC than other BC subtypes (52). Most TNBC cell lines, including MDA-MB-231 and Hs578T used in our study, are p53 mutated (53). In this context, the PD-L1–mediated inhibition of PTP1B in these TNBC cells is more likely tumor promoting, which may recapitulate the tumor-suppressing role of PTP1B in the immune system that normally expresses high levels of PD-L1. Our data suggest that PD-L1 protects the activated p38-MAPK from being dephosphorylated by PTP1B in TNBC cells and preserves p38-MAPK activity stimulated by conventional extracellular signals in tumor environment. It was shown that p38 activity is necessary for tumor progression by promoting the production of growth factors and cytokines that are necessary for tumor cell colonization and angiogenesis (54). Thus, the prolonged p38-MAPK activity in PD-L1–expressing TNBC tumors would facilitate tumor growth and metastasis.

More than increasing cell mobility, the EMT is a dedifferentiation program that enhances cell survival against environmental stresses and potentiates cancer stem cell generation, which both contribute to the development of metastasis and treatment resistance. Our study dissected the tumor-intrinsic PD-L1 pathway comprising PTP1B, p38-MAPK, GSK3β, and Snail that physically links PD-L1 to the regulation of the EMT status of TNBC cells. Moreover, it is plausible that PD-L1–mediated activation of p38-MAPK could be synergistic with the activation of conventional tumor-promoting signals like EGF and can be further activated by PD-1 expressed in the tumor-associated microenvironment. Consequently, PD-L1–expressing TNBC cells are dependent on PD-L1 to maintain highly aggressive behaviors, such as migration/invasion, anchorage-independent growth, metastasis, and chemoresistance. Indeed, we reported recently that PD-L1 desensitized melanoma and TNBC to chemotherapies (55). Our findings are in line with other studies showing that PD-1 by binding to PD-L1 increased the metastasis and chemoresistance of TNBCs (56, 57) via a PD-1-dependent increase of multidrug resistance 1 protein, whose expression is regulated by Snail (58, 59). Collectively, our results provide a molecular explanation for these observations and shed a light on a path for the management of metastasis and chemoresistance of PD-L1–expressing cancer cells.

Recently, a novel function of PD-L1’s intracellular domain in DNA damage response by stabilizing certain RNAs was reported in tumor cells (43). We observed a substantial loss of Snail mRNAs in PD-L1–knockout MDA-MB-231 cells but not in PD-L1–knockdown cells treated with siRNA. This suggests that the acute consequence of losing the tumor-intrinsic PD-L1 function is the increased destruction of Snail proteins, whereas the long-term or permanent loss of PD-L1 could further trigger the degradation of Snail mRNAs and achieve a more stable interruption of the EMT status and EMT-associated aggressiveness, such as metastasis and treatment resistance. Moreover, we found that the tumor-intrinsic PD-L1 signaling could be activated by PD-1, suggesting that PD-L1 functions as a receptor and PD-L1 antibodies may also alternate PD-L1 tumor-intrinsic functions. However, this concept has not been considered in current therapeutics involving PD-1/PD-L1 blockade. Our group identified one PD-L1 antibody (H1A) that promotes PD-L1 degradation and sensitizes TNBC cells to chemotherapy and γ-radiation (43). Our current results confirmed that H1A attenuated PD-L1 signaling in TNBC cells and suppressed tumor progression. Thus, it is necessary to characterize the effects of PD-L1 antibodies on PD-L1 degradation and PD-L1 tumor-intrinsic signaling for better therapeutic potential.

In summary, our study extends the understanding of PD-L1 biology in tumor cells and illustrates nonimmune effects of PD-L1 blockade therapies. One of these types of therapies, H1A, indeed exhibited substantially strong synergistic effect on suppressing TNBC progression in immunocompetent mice when combined with immune checkpoint blockade reagent. These findings will inspire and facilitate the development of novel, rational therapeutic approaches that can interfere with both tumor-extrinsic and tumor-intrinsic functions of PD-L1 in PD-L1–expressing TNBCs.

## Methods

### Cell lines and constructs.

MDA-MB-231 and Hs-578T cells were purchased from ATCC. PD-L1–knockout MDA-MB-231 cell lines were generated previously (55). E0771 cells were provided by Robin L. Anderson at Olivia Newton-John Cancer Research Institute (Heidelberg, Australia). Cells were maintained in DMEM supplemented with 10% FBS, 100 U/mL penicillin, and 100 μg/mL streptomycin in a humidified atmosphere at 37°C with 5% CO_2_.

Human PD-L1 cDNA was subcloned into pcDNA3 or pLVX3, provided by Zhenkun Lou at Mayo Clinic (Rochester, Minnesota, USA). pJ3H PTP1B was purchased from Addgene (plasmid 8601). Full-length or truncated PTP1B cDNAs were subcloned into pGEX-4T1 for GST fusion proteins. PD-L1 cytoplasmic domain cDNA (amino acids 262–290) was subcloned into pET-His6 (Addgene plasmid 29656).

### Lentivirus and transfection.

HEK293T cells (ATCC) were cotransfected with pLVX3-hPD-L1 (gift from Zhenkun Lou, Mayo Clinic, Rochester, Minnesota, USA), psPAX2 (Addgene plasmid 12260), and pMD2.G (Addgene plasmid 12259). Forty-eight and 72 hours later, culture medium was collected and lentivirus was concentrated using Lenti-X Concentrator (Clonetech Laboratories, Inc). MDA-MB-231 cells (5 × 10^5^) were incubated with lentivirus for 48 hours followed by puromycin (5 μg/mL) selection for another 3–5 days. Lipofectamine RNAiMax (Invitrogen, Thermo Fisher Scientific) or X-tremeGENE 9 (Roche) was used for expression of siRNA or exogenous proteins.

### RNAi and gene editing.

Predesigned, stealth RNAi negative control, and guide RNA (gRNA) siRNAs were purchased from Invitrogen, Thermo Fisher Scientific. Human PD-L1 siRNA sequences were 5′-GATATTTGCTGTCTTTATA-3′ and 5′-AAGGACTCACTTGGTAATTCT-3′. To knock out *Cd274* from E0771 cells, we used CRISPR/Cas9 following published procedure (55) with a gRNA for mouse PD-L1 (UCCACCACGUACAAGUCCUU).

### Antibodies and reagents.

The homemade mouse anti–human PD-L1 monoclonal antibodies, clone H1A and clone B11 (55, 60), and the hamster anti–mouse PD-1 monoclonal antibody clone G4 (42) were produced by the Antibody Hybridoma Core facility at Mayo Clinic. Antibodies purchased from Cell Signaling Technology are Snail (3879), Slug (9585), ZEB-1 (3396), β-catenin (8480), vimentin (5741), N-cadherin (13116), Claudin-1 (13255), β-TrCP (4394), PD-L1 (13684), phosphorylated p38 MAPK (4511), p38 MAPK (9212), phosphorylated ERK1/2 (9101), ERK1/2 (9102), phosphorylated Akt (9271), Akt (4691), T390-phosphorylated GSK3β (3458), S9-phosphorylated GSK3β (9336), GSK3β (9315), phosphorylated p70S6K (9205), and p70S6K (9202). We purchased the following primary antibodies: E-cadherin (BD Biosciences, 610181), ubiquitin (MilliporeSigma, MAB1510), PTP1B (Abcam, ab201974), HA-tag (MilliporeSigma, H9658), β-actin (MilliporeSigma, A3854), His-tag (Proteintech, 66005-1), CD68 (Abcam, ab125212), PD-1 (MilliporeSigma, MABC1132), and Alexa Fluor 488–conjugated goat anti-mouse antibody (Molecular Probes, Thermo Fisher Scientific). Anti-hamster Ig HRP detection kit was from BD Biosciences.

The following reagents were used: recombinant human and mouse IFN-γ (BioLegend), recombinant human PD-1 extracellular domain (G&P Biosciences), p38-MAPK inhibitor SB203580 (Cell Signaling Technology), proteasome inhibitor MG132 (MilliporeSigma), PTP1B inhibitor 3-(3,5-dibromo-4-hydroxybenzoyl)-2-ethyl-N-[4-[(2-thiazolylamino)sulfonyl]phenyl-6-benzofuransulfonamide (Santa Cruz Biotechnology, 765317-72-4), N-ethylmaleimide (MilliporeSigma), GSK3β inhibitor SB216763 (Selleckchem), and iodoacetamide (MilliporeSigma).

### Cell proliferation.

Cell proliferation was determined by MTT assay. Cells (20,000/well) were plated in 96-well microtiter plates and cultured for 48 or 72 hours. Viable cells in each well were then measured.

### Soft agar colony formation.

First, 0.6% base agar in complete medium was added to a 6-well plate (1 mL/well) and allowed to solidify at room temperature. Cells (5000/well) mixed with 1 mL 0.4% agarose in complete medium were added on top of the base agar and allowed to solidify at room temperature. After 3-week culture, colonies were stained with MTT and scanned with GelCount colony counter. Numbers of colonies were quantified using GelCount software.

### Cell migration.

In vitro cell migration assay was performed using modified Boyden chamber (Neuroprobe) equipped with type I collagen–coated (10 μg/mL) membrane. Then, 20,000 serum-starved cells were added to the upper chamber in serum-free medium with complete medium containing 10% FBS in the lower chamber. Four hours later, cells migrating to the underside of the membrane were fixed and stained with DAPI. For quantification of migrated cells on each membrane, cell numbers in 5 randomly selected, nonoverlapping fields were counted under microscope and averaged. Each sample was performed in duplicates. Each experiment was independently repeated 3 times.

### Quantitative real-time PCR.

Total RNA was isolated from cells using the Qiagen RNeasy kit (Qiagen). cDNA was then obtained from 1 μg of total RNA using cDNA reverse transcription kit (Applied Biosystems, Thermo Fisher Scientific). Quantitative real-time PCR was performed using SYBR Green PCR Master Mix with CFX384 Real-Time system (Bio-Rad) and analyzed using CFX Manager software (Bio-Rad). Primers were: E-cadherin-forward, 5′-CGAGAGCTACACGTTCACGG-3′; E-cadherin-reverse, 5′-GGGTGTCGAGGGAAAAATAGG-3′; Snail-forward, 5′-TCGGAAGCCTAACTACAGCGA-3′; Snail-reverse, 5′-AGATGAGCATTGGCAGCGAG-3′; GAPDH-forward, 5′-GAAGGTGAAGGTCGGAGTC-3′; GAPDH-reverse, 5′-GAAGATGGTGATGGGATTTC-3′.

### Flow cytometry.

Fluorochrome-conjugated antibodies against human B7-H1 (M1H1), PD-1 (EH12.2H7), and CD80 (L307.4) were purchased from BD Biosciences, BioLegend, and eBioscience (Thermo Fisher Scientific), respectively. Cells were labeled with fluorochrome-conjugated antibodies in PBS with 2% FBS and subjected to flow cytometry analysis using FlowJo software (Tree Star).

### Immunoblotting.

Cells were lysed in 1× SDS loading buffer, and protein samples were separated on 7.5% or 10% SDS-PAGE, transferred to a PVDF membrane, and subjected to immunoblotting as described (61). Bands were visualized and imaged using ChemiDoc Touch image system (Bio-Rad). See complete unedited blots in the supplemental material.

### Immunofluorescence microscopy.

Indirect immunofluorescence microscopy was performed as described previously (61). Cover glass was mounted using mounting buffer (Vector Laboratories) and observed under Nikon Eclipse fluorescence microscope.

### Immunoprecipitation.

Immunoprecipitation was performed as described (61). To detect Snail ubiquitination, cells were lysed as described (62) for denaturing immunoprecipitation.

### GST pull-down.

GST, the GST-fused PTP1B (amino acids 1–277, 1–321, and full length), and His-PDL1-CT were expressed in *E*. *coli* and purified using glutathione or His resin, respectively. GST pull-down assay was performed as described (61) using purified His-PDL1-CT (0.5 μg) and 0.5 μg GST or GST-fusion proteins.

### Phosphatase activity.

GST, MBP, and MBP-fused PD-L1 cytoplasmic domain (MBP-PDL1-CT) were purified from *E*. *coli*. PTP1B activity was measured using PTP1B Colorimetric Assay Kit (BPS Bioscience, 30019). Absorbance at 415 nm of each sample was determined by a microplate reader (Synergy H1 microplate reader, BioTek) for 1 hour with 5-minute interval at room temperature. The slope of absorbance increase was calculated to represent the activity of PTP1B.

### TNBC mouse models.

NOD/SCID and C57BL/6 mice (The Jackson Laboratory) were maintained in specific pathogen–free and regular rodent facilities, respectively, under controlled light and humidity with normal food and water supply. MDA-MB-231 or E0771 cells were implanted subcutaneously into the mammary fat pad of female NOD/SCID mice or C57BL/6 mice. Tumor volume (V) was measured with calipers and calculated by using the standard formula V = 0.5 × LW^2^ (L, length; W, width). For experimental lung metastases, 8 × 10^5^ cells in 100 μL sterile PBS were injected into the tail vein. At the endpoint when the body condition score reached 1, primary tumors and lungs were excised promptly after euthanasia and fixed with 10% formaldehyde solution, then embedded in paraffin for further processing and analyses. Control mouse or hamster IgG, hamster anti–mouse PD-1 antibodies, and/or homemade mouse anti–human PD-L1 antibodies (clone H1A or B11) were administrated via intraperitoneal injection.

### Histological analysis.

Hematoxylin and eosin staining was carried out following standard protocol. For immunohistochemistry staining, tissue sections (5 μm thickness) were deparaffinized in xylene and rehydrated through alcohol gradient, incubated with hydrogen peroxide (Dako) for 10 minutes to quench the endogenous peroxidase activity, and then rinsed with PBS 3 times. Antigen retrieval was performed using a steamer for 30 minutes in 0.1 M citrate buffer (pH 6.0). Serum-free protein block (Dako) was used to prevent nonspecific protein binding. Sections were then incubated with primary antibodies overnight at 4°C followed by 60-minute incubation with anti-rabbit secondary antibody (111-035-144, Jackson ImmunoResearch) at room temperature. The slides were developed with DAB Peroxidase Substrate Kit (Dako), counterstained with hematoxylin, dehydrated in ethanol, cleared in xylene, and then mounted with Permount mounting medium (Thermo Fisher Scientific).

### Statistics.

Statistical comparisons were made using an unpaired 2-tailed Student’s *t* test (in vitro studies) or 1-way ANOVA analysis (in vivo studies) with GraphPad Prism 8 software. When multiple comparison was applied, *P* value from *t* test was adjusted with Bonferroni’s method to control the type 1 error in the 5% level. In cases where multiple groups were compared but not to a single control group, we used 1-way ANOVA analysis with the *P* value adjusted by Tukey’s HSD using R function “aov” and “TukeyHSD” from package “stats” in R version 3.6.3. *P* value less than 0.05 was considered statistically significant. All data represented in plots represent the mean ± SEM. All in vitro assays were performed in biological triplicates.

### Study approval.

Animal studies were carried out following guidelines and protocols approved by the Institutional Animal Care and Use Committee at Mayo Clinic.

## Author contributions

KL and HD conceived and designed the study. CC and SL developed methodology. CC, SL, JX, MQ, XL, and YH acquired data (provided animals, antibodies, purified proteins, facilities, etc.). CC, SL, JX, MQ, XL, and KL analyzed and interpreted data (e.g., statistical analysis, computational analysis). CC, SL, JH, HD, and KL wrote, reviewed, and/or revised the manuscript. YH and XL provided administrative, technical, or material support. HD and KL supervised the study.

## Supplementary Material

Supplemental data

## Version 1 04/22/2021

Electronic publication
