# Supplementary material for: PD-L1 tumor-intrinsic signaling and its therapeutic implication in triple-negative breast cancer
Source: JCI Insight. 2021 Apr 22;6(8):e131458. doi: 10.1172/jci.insight.131458 (PMC8119208; doi:10.1172/jci.insight.131458)

Supplemental Information

PD-L1 Tumor-Intrinsic Signaling and Its Therapeutic Implication in Triple-Negative Breast Cancer

Chunhua Chen[1], Shiheng Li[1,†], Junli Xue[1,†], Manlong Qi[1,†], Xin Liu[2], Yan Huang[1,3], Jinghua Hu[1,3], Haidong Dong[2,*], Kun Ling[1,*]

[1] *Department of Biochemistry and Molecular Biology, Mayo Clinic, 200 First Street SW, Rochester, MN 55902, USA;*

[2] *Departments of Urology and Immunology, Mayo Clinic, 200 First Street SW, Rochester, MN 55902, USA;*

[3] *Department of Nephrology and Hypertension, Mayo Clinic, 200 First Street SW, Rochester, MN 55902, USA.*

[†] *Current address: S. L., Department of Breast Surgery, The First Hospital of Jilin University, 71 Xinmin Street, Changchun, Jilin Province, China; J. X., Department of Oncology, Shanghai East Hospital, Tongji University School of Medicine, 150 Ji-Mo Road, Shanghai 200120, China; M. Q., Department of Clinical Genetics, Shengjing Hospital, China Medical University, 16 Pu-He Boulevard, Shenyang 110004, China.*

* C*orrespondence*: Kun Ling, Department of Biochemistry and Molecular Biology, Cancer Center, Mayo Clinic, Guggenheim 16-04, 200 First Street SW, Rochester, MN 55902, USA. Tel: 507-293-3498, FAX: 507-266-9315, ling.kun@mayo.edu. Haidong Dong, Department of Immunology, Mayo Clinic, Guggenheim 4-01B, 200 First Street SW, Rochester, MN 55902, USA. Tel: 507-284-5482, dong.haidong@mayo.edu

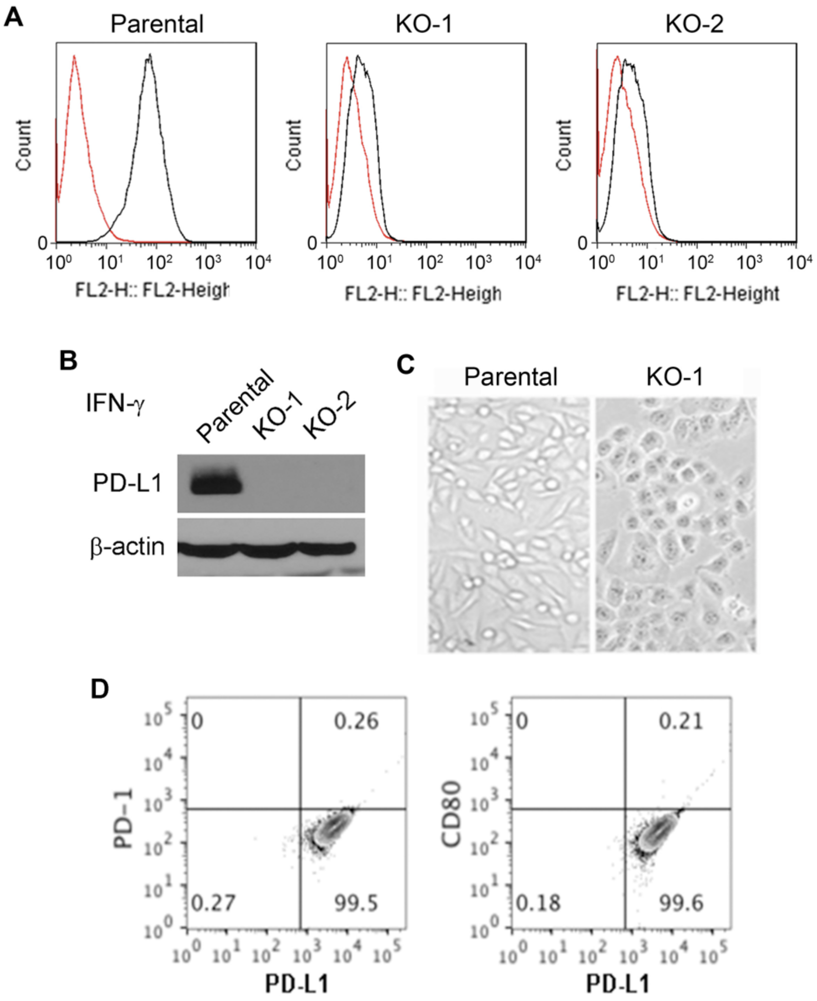

Supplemental Figure 1

Characterization of PD-L1-null MDA-MB-231 cells. PD-L1 specific guide-RNAs were introduced in MDA-MB-231 cells for the CRISPR/Cas9 mediated gene editing. Single clones KO-1 and KO-2 were obtained and characterized. (**A**) Flow cytometry analysis confirmed that KO-1 and KO-2 cells do not express PD-L1. (**B**) IFN-γ cannot induce PD-L1 expression in KO-1 and KO-2 cells. Parental, KO-1, and KO-2 MDA-MB-231 cells were treated with 10 ng/ml IFN-γ for 48 hours. Cells were then lysed and analyzed by immunoblotting using indicated antibodies. (**C**) PD-L1-null MDA-MB-231 cells showed epithelial-like morphology. Parental and KO-1 MDA-MB-231 cells were visualized by light microscope. (**D**) MDA-MB-231 cells do not express PD-1 or CD80, two natural PD-L1 receptors. Parental MDA-MB-231 cells were double labeled with FITC-conjugated PD-1 and PE-conjugated PD-L1 antibodies or PE-conjugated CD80 and Cy7-conjugated PD-L1 antibodies, and then subjected to flow cytometry analyses.

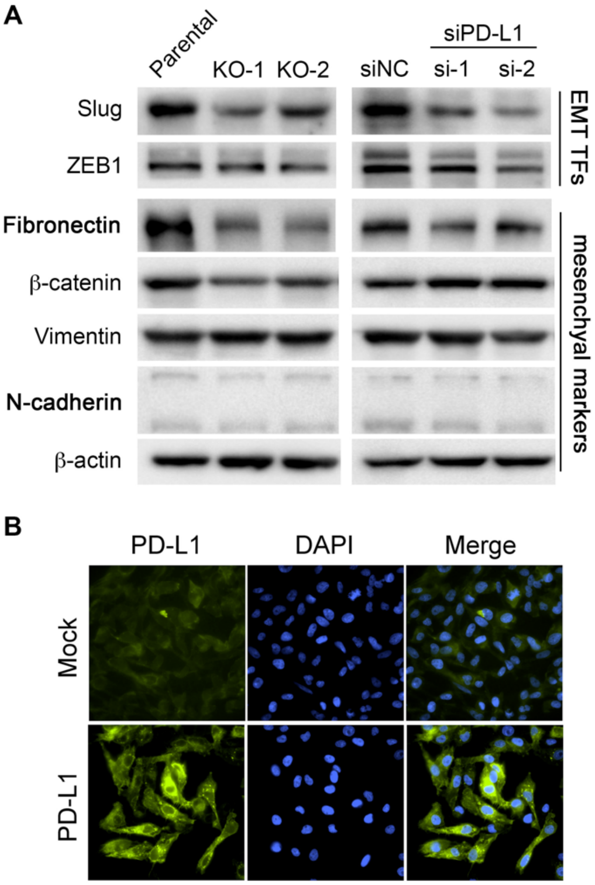

Supplemental Figure 2

PD-L1 deficiency partially reverses the EMT in MDA-MB-231 cells. (**A**) Slug and fibronectin were downregulated in PD-L1-deficient cells. Indicated EMT transcription factors and mesenchymal markers were determined by immunoblotting in control, PD-L1 null, or PD-L1 knockdown cells. (**B**) Lentivirus mediated expression of ectopic PD-L1 in MDA-MB-231 cells was highly efficient. MDA-MB-231 cells were transduced by lentivirus carrying PD-L1 expression construct for 48 hours. The expression efficiency was confirmed by immunofluorescence microscopy with anti-PD-L1 antibody. DAPI staining showed nucleus.

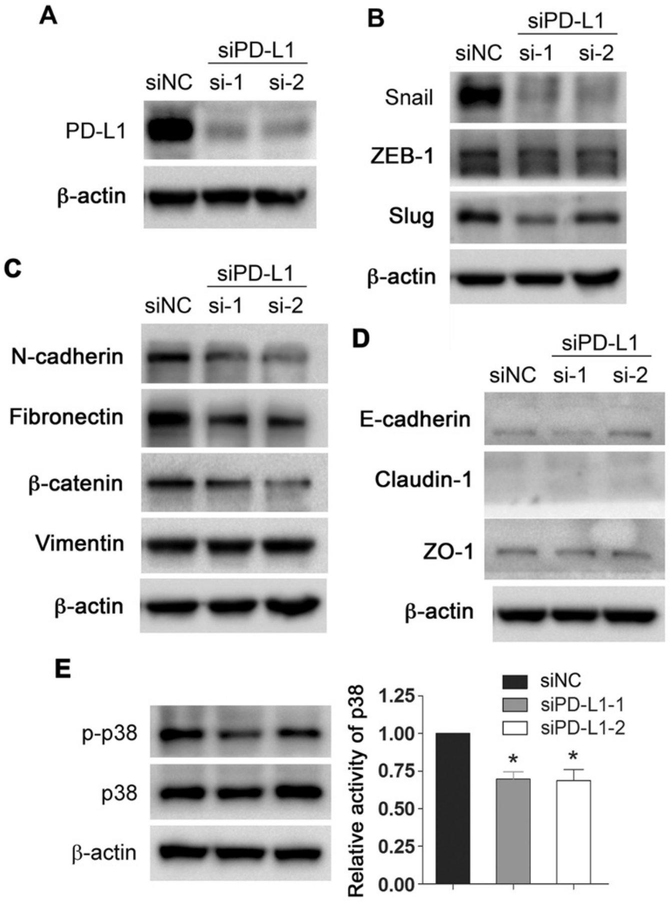

Supplemental Figure 3

PD-L1 is required for the maintenance of EMT status in Hs578T cells. Hs578T cells were transfected with control (siNC) or PD-L1 specific (si-1 and si-2) siRNAs for 48 hours. Cells were then lysed and analyzed by immunoblotting to determine the protein level of PD-L1 (**A**), EMT transcription factors (**B**), mesenchymal markers (**C**), epithelial markers (**D**), and activity of p38-MAPK (**E**). The activity of p38-MAPK in control or PD-L1 knockdown Hs578T cells were quantified and normalized against that in control cells. Results from three independent experiments were statistically analyzed and plotted. *, $P < 0.05$.

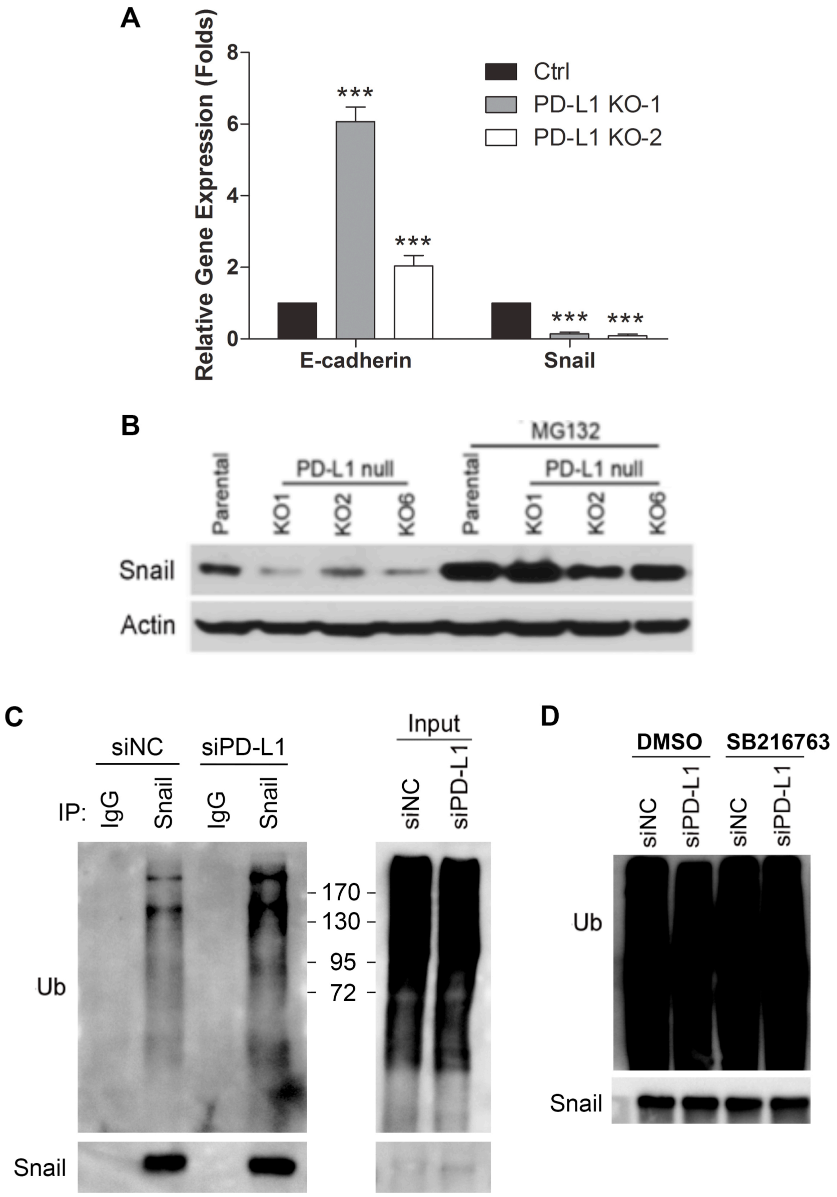

Supplemental Figure 4

PD-L1 protects Snail proteins from being destructed. (**A**) Stable, complete loss of PD-L1 suppressed the transcription of E-cadherin and Snail. mRNA levels of E-cadherin and Snail in parental or PD-L1-null MDA-MB-231 cells were determined by RT-qPCR. (**B**) Inhibition of proteasome restored Snail in parental and PD-L1-null MDA-MB-231 cells. Parental MDA-MB-231 cells and three PD-L1-null clones with or without 6-hour MG132 treatment (10 µM) were subjected to immunoblotting to determine Snail protein levels. (**C**) Endogenous Snail exhibited higher level of ubiquitination in PD-L1-depleted cells. After being transfected with control (siNC) or PD-L1 specific (siPD-L1, si-1) siRNAs for 48 hours, MDA-MB-231 cells were treated with MG132 for 6 hours, and then lysed for immunoprecipitation with normal mouse IgG or Snail-specific antibodies. Ubiquitination of precipitated proteins or in total cell lysates were determined by immunoblotting using indicated antibodies. Note that the total ubiquitination level in cell lysates was not changed in PD-L1-depleted cells. (**D**) This is a control blot for Figure 3F, showing that the global ubiquitination levels or Snail protein levels in whole cell lysates were not changed by PD-L1 depletion or SB216763 treatment.

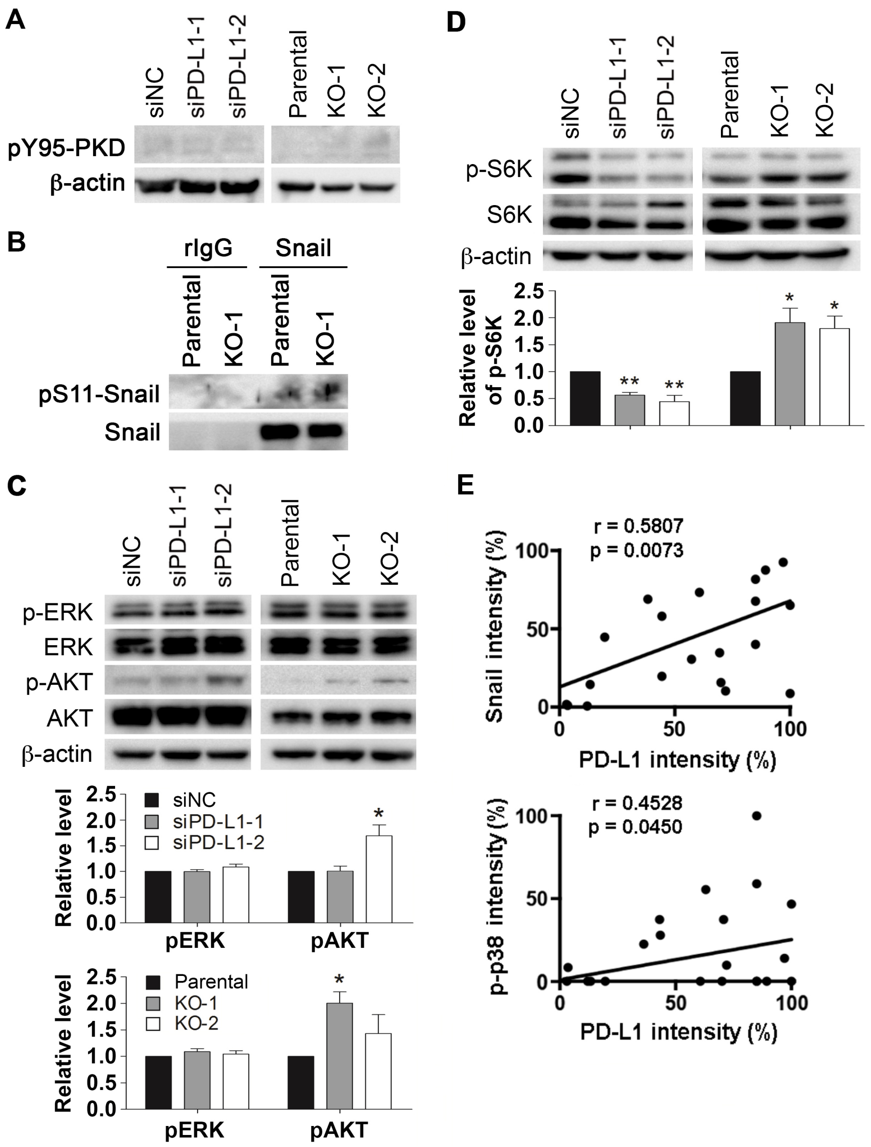

Supplemental Figure 5

Loss of PD-L1 has no effect on the activity of PKD, ERK, AKT, or p70-S6K. Cell lysates were prepared using parental, PD-L1-null (KO-1 and KO-2), control siRNA transfected (siNC), or PD-L1 siRNA transfected (siPD-L1-1 and siPD-L1-2) MDA-MB-231 cells. Cell lysates from each group were subjected to immunoblotting with indicated antibodies to determine the activity of PKD (**A**), Snail phosphorylation by PKD (**B**), ERK (**C**), and p70 S6K (**D**), respectively. The relative activity of ERK, AKT, or p70S6K were quantified by normalizing the phosphorylated ERK, AKT, or p70S6K to total ERK, AKT, or p70S6K, respectively. Results from three independent experiments were statistically analyzed and plotted. *, $P < 0.05$; **, $P < 0.01$. (**E**) Levels of Snail and activated p38-MAPK are both positively correlated with PD-L1 level in TNBC patients. Tissues from 20 deidentified, PD-L1-positive TNBC patients were withdrawn from the Tissue Registry at The First Hospital of Jilin University (Changchun, China), processed, and sliced. These tissue slides were then subjected to immunohistochemistry using Snail antibody, PD-L1 antibody, or phosphorylated p38 antibody. Staining intensity of each antibody in each patient tissue was scored and results were plotted as shown. Pearson Correlation Coefficient (r) and the *p* value were calculated and shown in each plot.

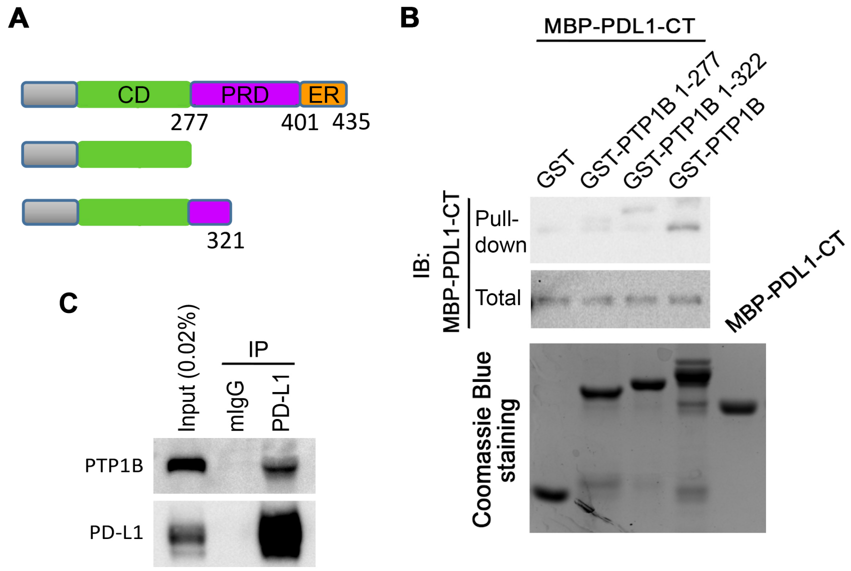

Supplemental Figure 6

PD-L1 directly interacts with PTP1B. (**A**) Structural domains of the full-length PTP1B and truncated variants. (**B**) The cytoplasmic domain of PD-L1 directly binds to the C-terminus of PTP1B. GST, GST-fused full-length or truncated (aa 1-277 and aa 1-321) PTP1B, and MBP-fused PD-L1 cytoplasmic domain (MBP-PDL1-CT) were expressed and purified from *E. coli*. GST pull-down assays were performed by incubating MBP-PDL1-CT with GST alone or GST-PTP1B proteins, 0.5 μg each. MBP-PDL1-CT pulled down by GST beads was visualized by immunoblotting. Lower panel showed purified proteins on SDS-PAGE visualized by Coomassie Blue staining. (**C**) PD-L1 forms complex with a small portion of PTP1B in non-stimulated TNBC cells cultured in tissue culture dishes.

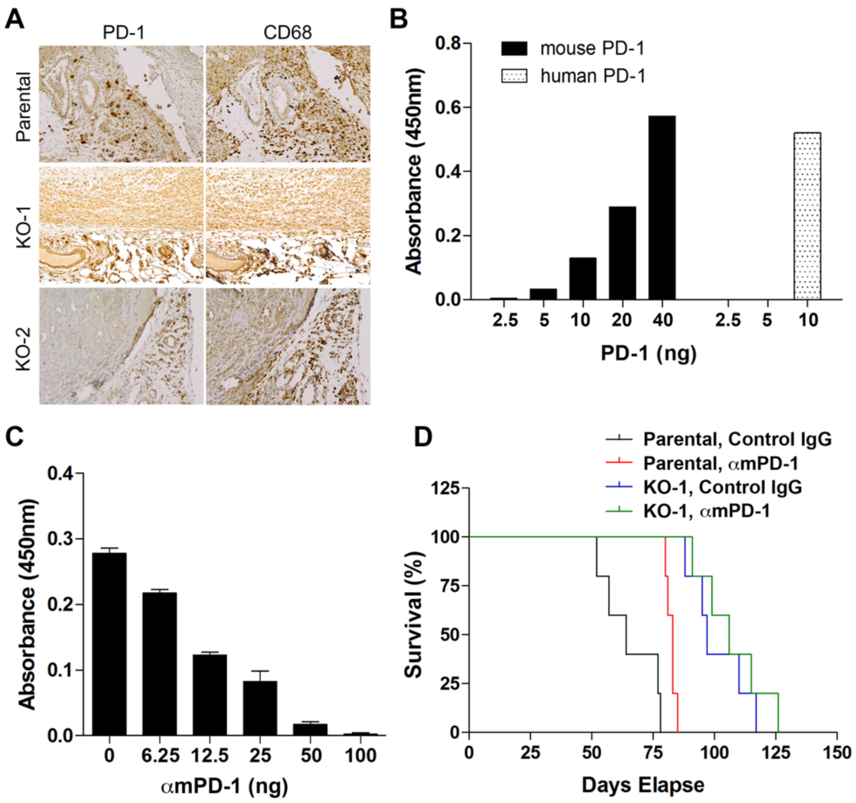

Supplemental Figure 7

PD-1 expressed in tumor microenvironment associates with the progression of MDA-MB-231 tumors via PD-L1 expressed in tumor cells. (**A**) Parental and PD-L1-null (KO-1 and KO-2) MDA-MB-231 tumors were collected from SCID mice on Day-52 after inoculation and processed for immunohistochemistry staining using antibodies against PD-1 or CD68. Power of eyepiece: 10×; power of objective: 20×. Note that the level of PD-1 expression and macrophage infiltration in tumor peripheral areas are comparable in parental and PD-L1-null tumors. (**B** and **C**) Mouse PD-1 binds to human PD-L1 and this binding is effectively interrupted by our hamster anti-mouse PD-1 antibody (αmPD-1). ELISA assays were performed using PD-1 (Biotinylated): PD-L1 Inhibitor Colorimetric Screening Assay Kit (BPS Bioscience, 72018) following manufacturer's instruction. Results from at least three independent experiments were statistically analyzed and plotted as mean ± S.E.M. (**B**) Indicated amount of biotinylated recombinant mouse or human PD-1 (BPS Bioscience) were added to human PD-L1 precoated 96-well plate for ELISA assay. (**C**) Biotinylated recombinant mouse PD-1 (20 ng) was incubated with indicated amount of αmPD-1 at room temperature for 20-min, then added to the 96-well plate precoated with human PD-L1 for ELISA assay. (**D**) PD-1 blocking antibody suppressed the progression of parental tumors but not PD-L1-null tumors. 2×10^6 parental or KO-1 MDA-MB-231 cells were injected into the mammary fat pad of female NOD/SCID mouse (10 mice per tumor type). On Day 4 after inoculation, five mice were treated with control hamster IgG and the other five were treated with αmPD-1 via intraperitoneal injection, 100 μg/mouse, twice/week for 6 weeks. Long-rank test supported significant difference between the survival of mice carrying parental tumors treated with control IgG and that of mice carrying parental tumors treated with αmPD-1 (P = 0.0018). However, there was no significant difference between the survival of mice carrying KO-1 tumors no matter whether they were treated with control IgG or αmPD-1 (P = 0.4723).

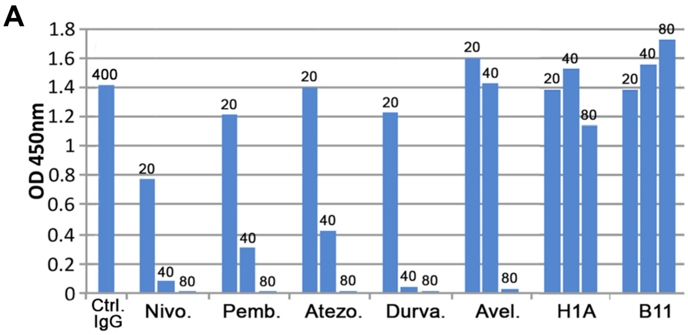

B

Antibody	k_a ($m^{-1}s^{-1}$)	k_d (s^{-1})	K_D (pM)	Mean R_{max}
Atezo	5.68×10^5	1.11×10^{-4}	195 ± 10	0.138 ± 0.041
H1A	5.36×10^5	9.78×10^{-5}	182 ± 10	0.130 ± 0.020
B11	4.32×10^5	2.67×10^{-4}	617 ± 4.4	0.114 ± 0.013

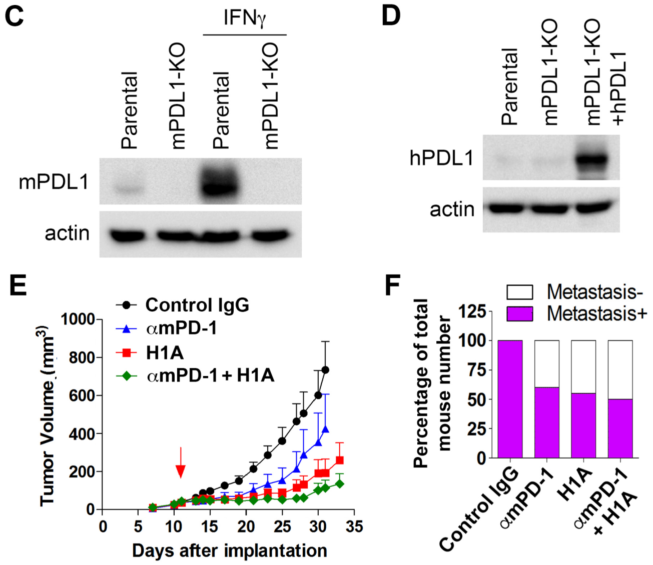

Supplemental Figure 8

Supplemental materials for Figure 8. (**A**) H1A and B11 cannot block PD-1 binding to PD-L1. ELISA results reflect the capacities of indicated antibodies to interrupt the binding of recombinant PD-1 with PD-L1 at different concentrations (20/40/80 ng). Control IgG, control mouse IgG; Nivo, nivolumab; Pemb, pembrolizumab; Atezo, atezolizumab; Durva, durvalumab. (**B**) H1A and B11 possess high affinity to bind to PD-L1. Binding kinetics of indicated antibodies to PD-L1 ectodomain were calculated from ELISA results. (**C**) E0771 cells were subjected to CRISPR/Cas9-mediated gene editing to inactivate *Cd274* that encodes mouse PD-L1. The resulted clone (mPDL1-KO) showed no PD-L1 expression with or without mouse IFNγ treatment (100 ng/mL for 48-hr). (**D**) Stable expression of human PD-L1 in E0771-mPDL1-KO cells. E0771-mPDL1-KO cells were infected with lentivirus carrying human PD-L1 cDNA for 48 hours and then treated with puromycin (5 μg/ml) for 5 days to ensure the stable expression of human PD-L1 in these cells. (**E** and **F**) 1×10^6 PD-L1-humanized E0771 cells were injected into the mammary fat pad of female C57BL/6 mice. On Day-11 after inoculation (red arrow), mice were treated with 200 μg control IgG, hamster anti-mouse PD-1 (αmPD-1), mouse anti-human PD-L1 antibody H1A, or αmPD-1 combined with H1A (100 μg αmPD-1 + 100 μg H1A) intraperitoneally once every 3 days for total 5 times (9-10 mice/group). Animals were terminated when the body condition score reached 1 or on Day-75 after tumor inoculation. (**E**) Combined treatment of αmPD-1 and H1A showed the strongest suppression on tumor growth. Tumors were measured by calipers weekly. Tumor volume was calculated by using the equation $V = 0.5$

× LW^2. (**F**) Combined treatment of αmPD-1 and H1A showed the strongest suppression on lung metastasis. Lungs were collected when animal was terminated and processed for H&E staining to evaluate lung metastasis.

Fig.1

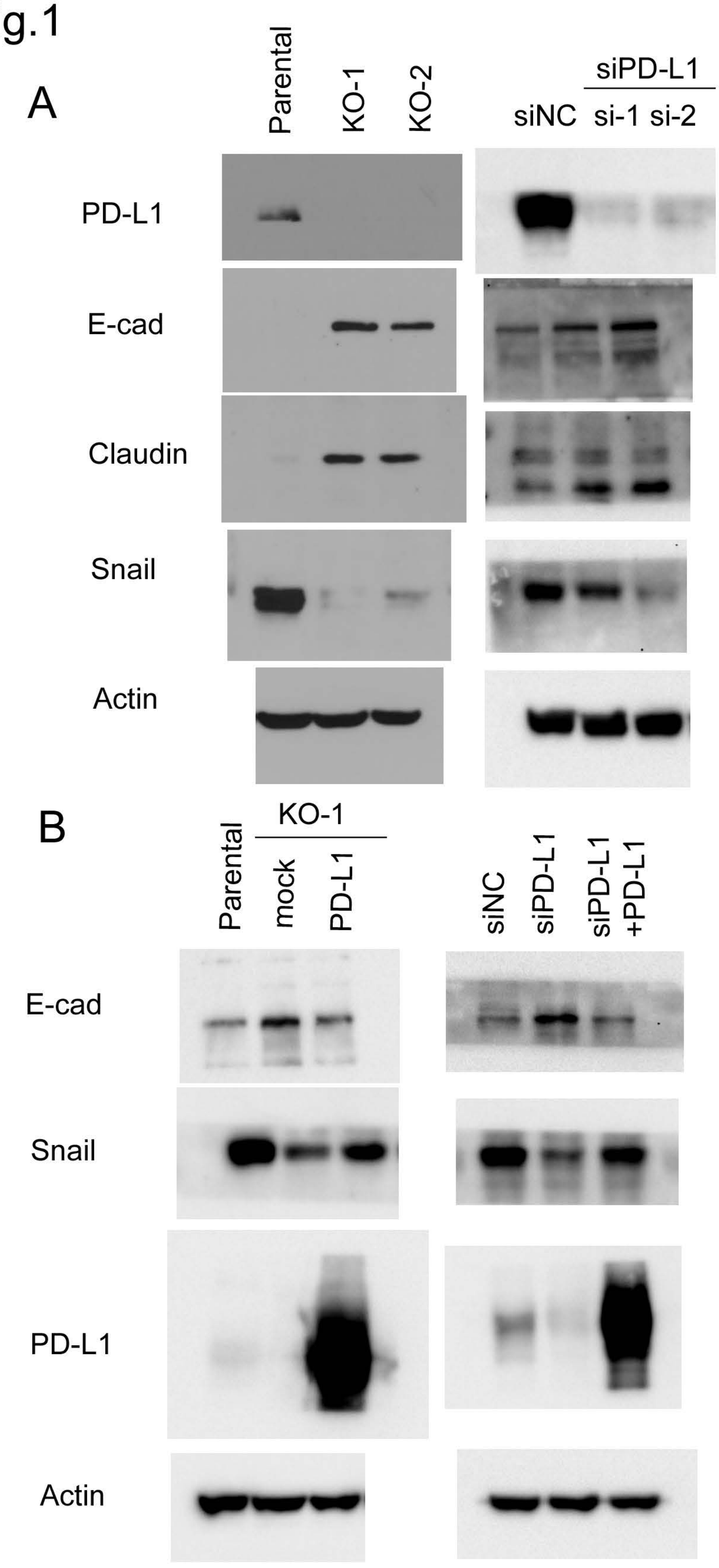
A
Parental
KO-1
KO-2
siPD-L1
siNC
si-1
si-2
PD-L1
E-cad
Claudin
Snail
Actin
B
KO-1
Parental
mock
PD-L1
siNC
siPD-L1
siPD-L1 +PD-L1
E-cad
Snail
PD-L1
Actin

Fig.3

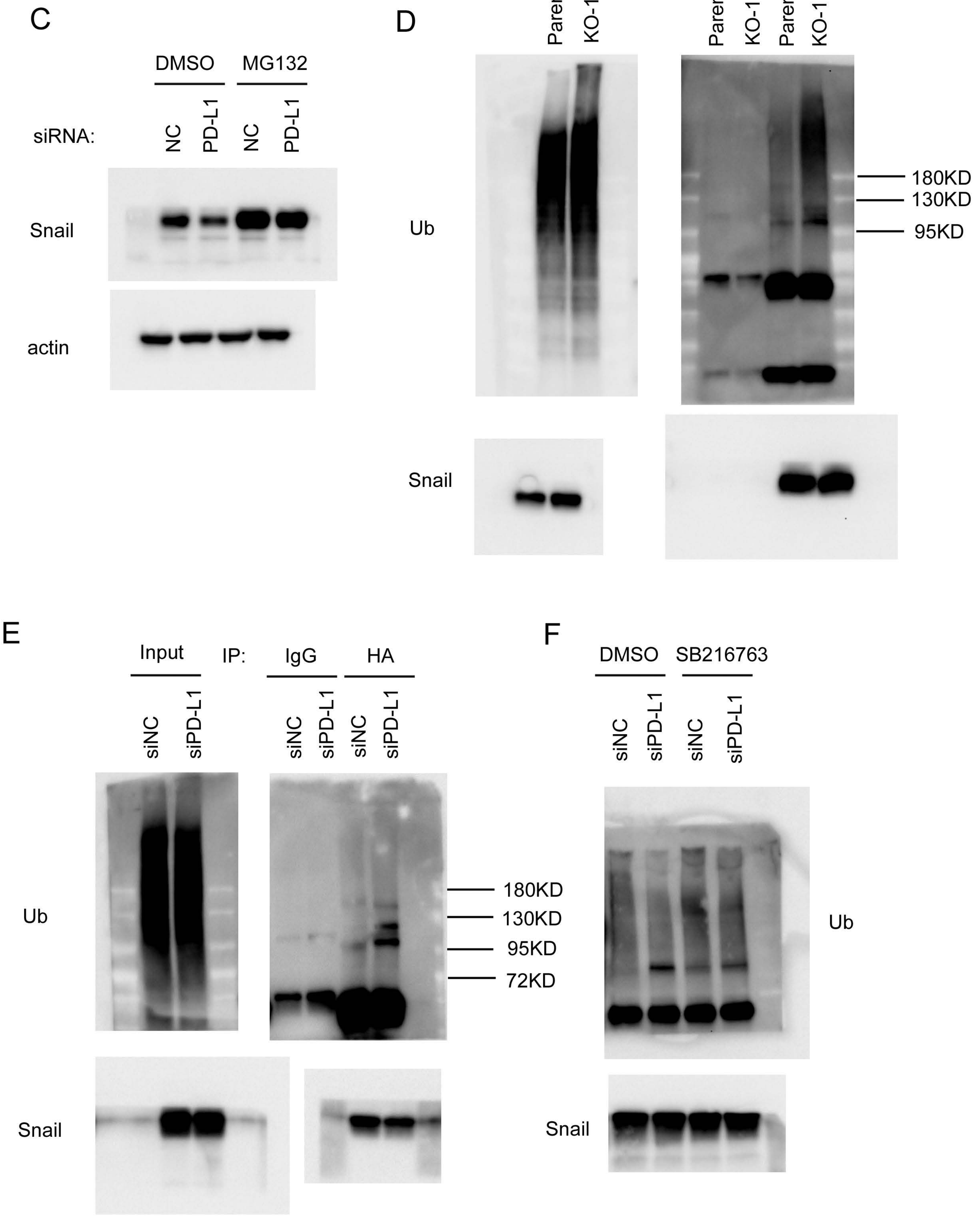
C
DMSO
MG132
siRNA:
NC
PD-L1
NC
PD-L1
Snail
actin
D
Input
IP:
IgG
HA
Parental
KO-1
Parental
KO-1
Parental
KO-1
Ub
180KD
130KD
95KD
Snail
E
Input
IP:
IgG
HA
siNC
siPD-L1
siNC
siPD-L1
siNC
siPD-L1
Ub
180KD
130KD
95KD
72KD
Snail
F
DMSO
SB216763
siNC
siPD-L1
siNC
siPD-L1
Ub
Snail

Fig.4

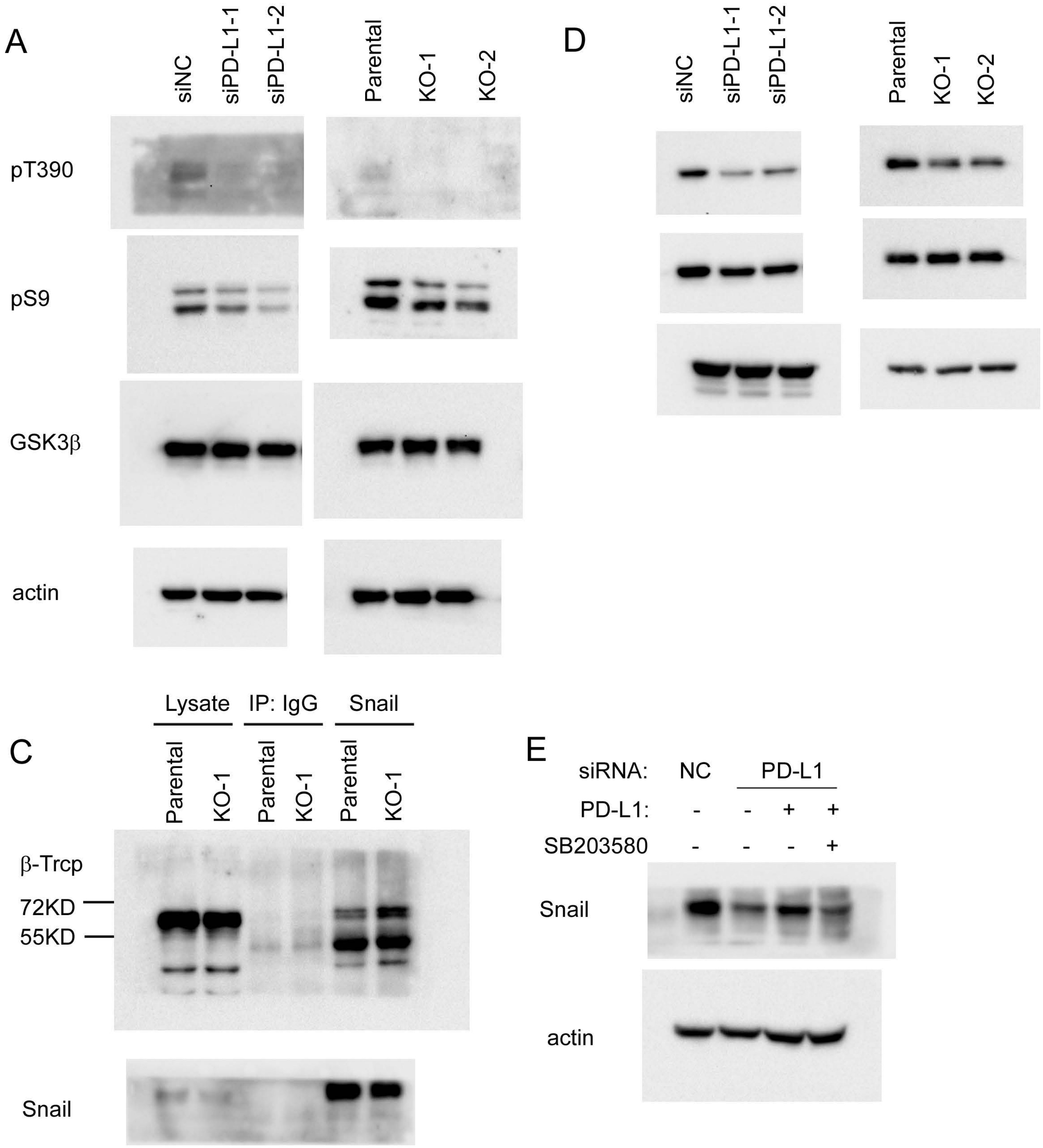
A
siNC
siPD-L1-1
siPD-L1-2
Parental
KO-1
KO-2
pT390
pS9
GSK3β
actin
D
siNC
siPD-L1-1
siPD-L1-2
Parental
KO-1
KO-2
C
Lysate
IP: IgG
Snail
Parental
KO-1
Parental
KO-1
Parental
KO-1
β-Trcp
72KD
55KD
Snail
E
siRNA:
NC
PD-L1
PD-L1:
-
-
+
+
SB203580
-
-
-
+
Snail
actin

Fig. 5

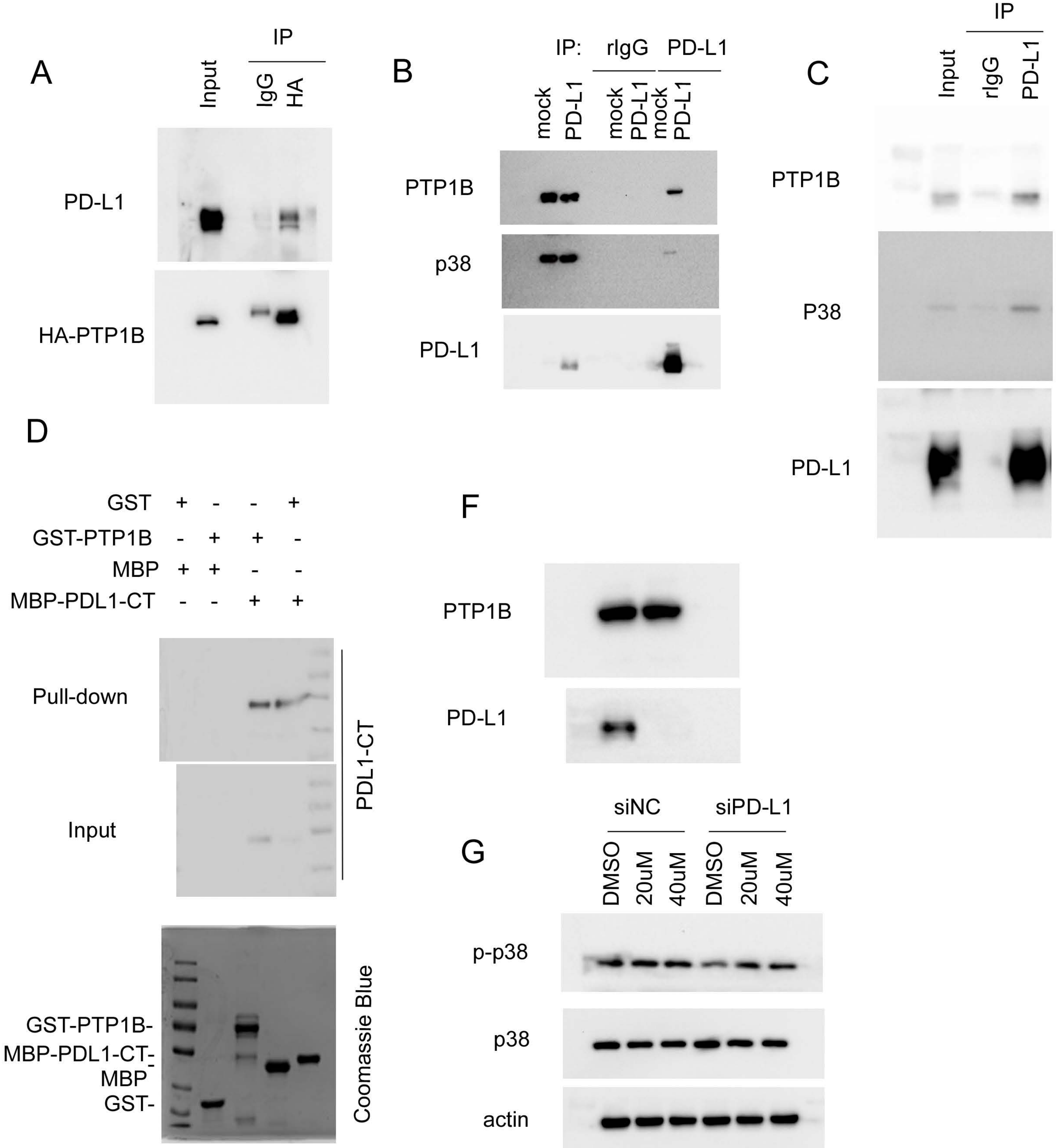
A
Input
IP
IgG
HA
PD-L1
HA-PTP1B
B
IP:
rIgG
PD-L1
mock
PD-L1
mock
PD-L1
mock
PD-L1
PTP1B
p38
PD-L1
C
IP
Input
rIgG
PD-L1
PTP1B
P38
PD-L1
D
GST + - - +
GST-PTP1B - + + -
MBP + + - -
MBP-PDL1-CT - - + +
Pull-down
Input
PDL1-CT
GST-PTP1B-
MBP-PDL1-CT-
MBP-
GST-
Coomassie Blue
F
PTP1B
PD-L1
G
siNC
siPD-L1
DMSO
20uM
40uM
DMSO
20uM
40uM
p-p38
p38
actin

Fig.6

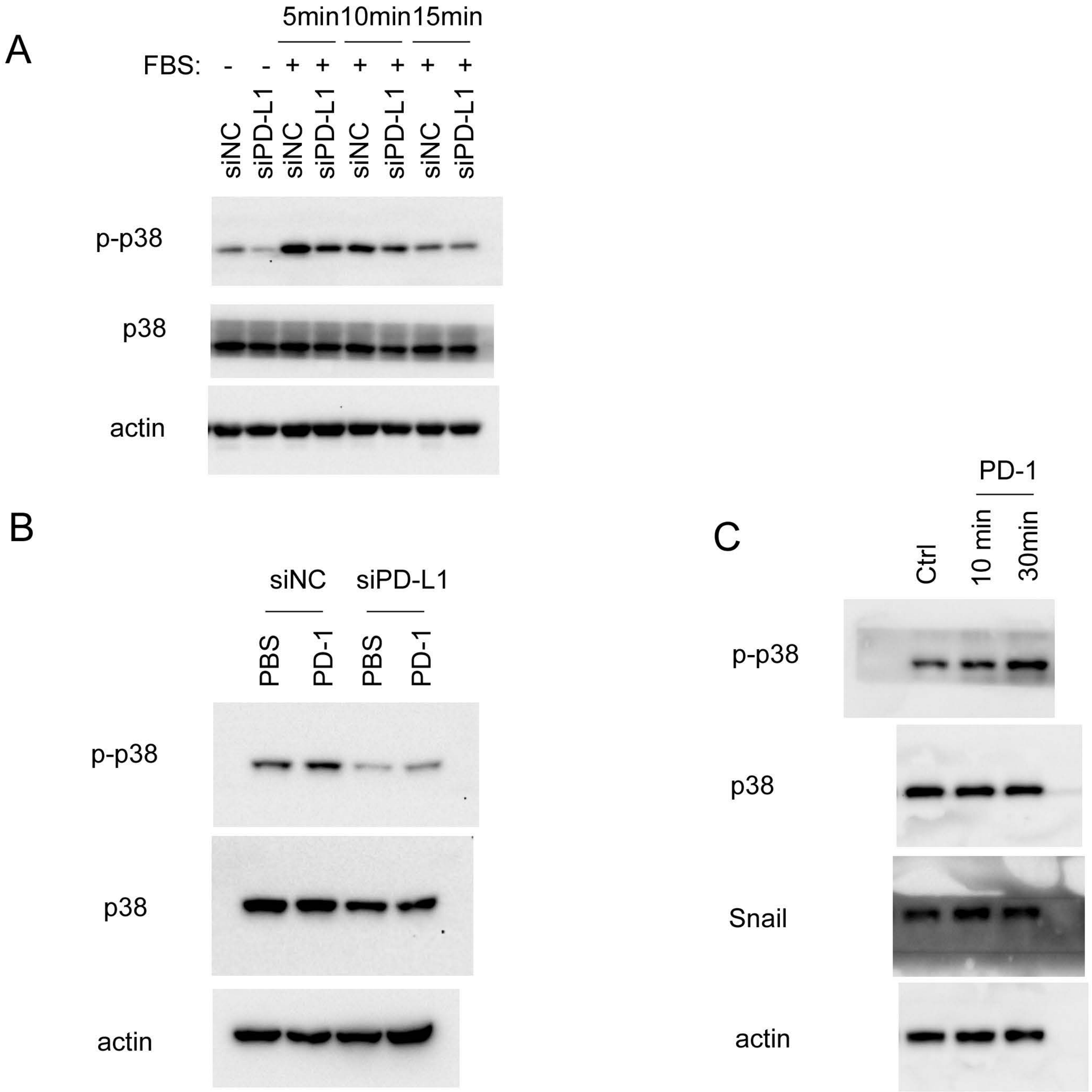
A
5min
10min
15min
FBS: - - + + + + + +
siNC
siPD-L1
siNC
siPD-L1
siNC
siPD-L1
siNC
siPD-L1
p-p38
p38
actin
B
siNC
siPD-L1
PBS
PD-1
PBS
PD-1
p-p38
p38
actin
C
PD-1
Ctrl
10 min
30min
p-p38
p38
Snail
actin

Fig 7

A

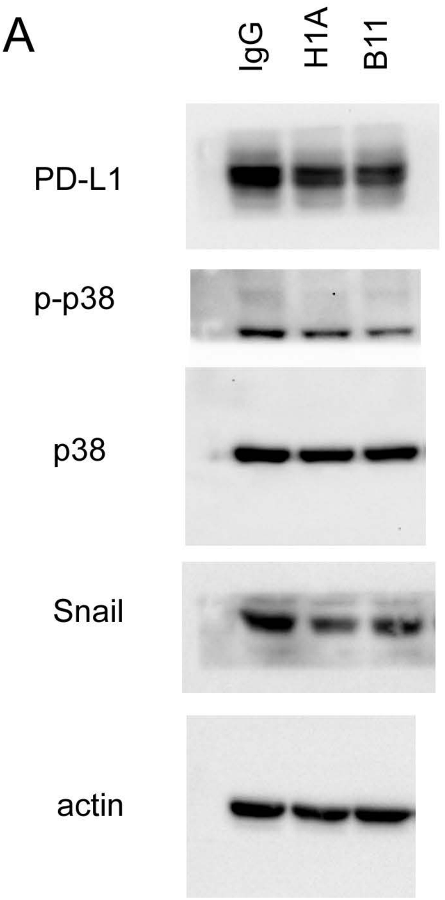

Supplementary Fig 1

B

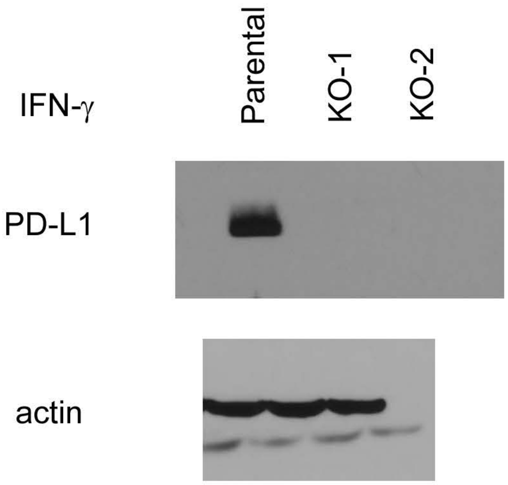

Supplementary Fig. 2

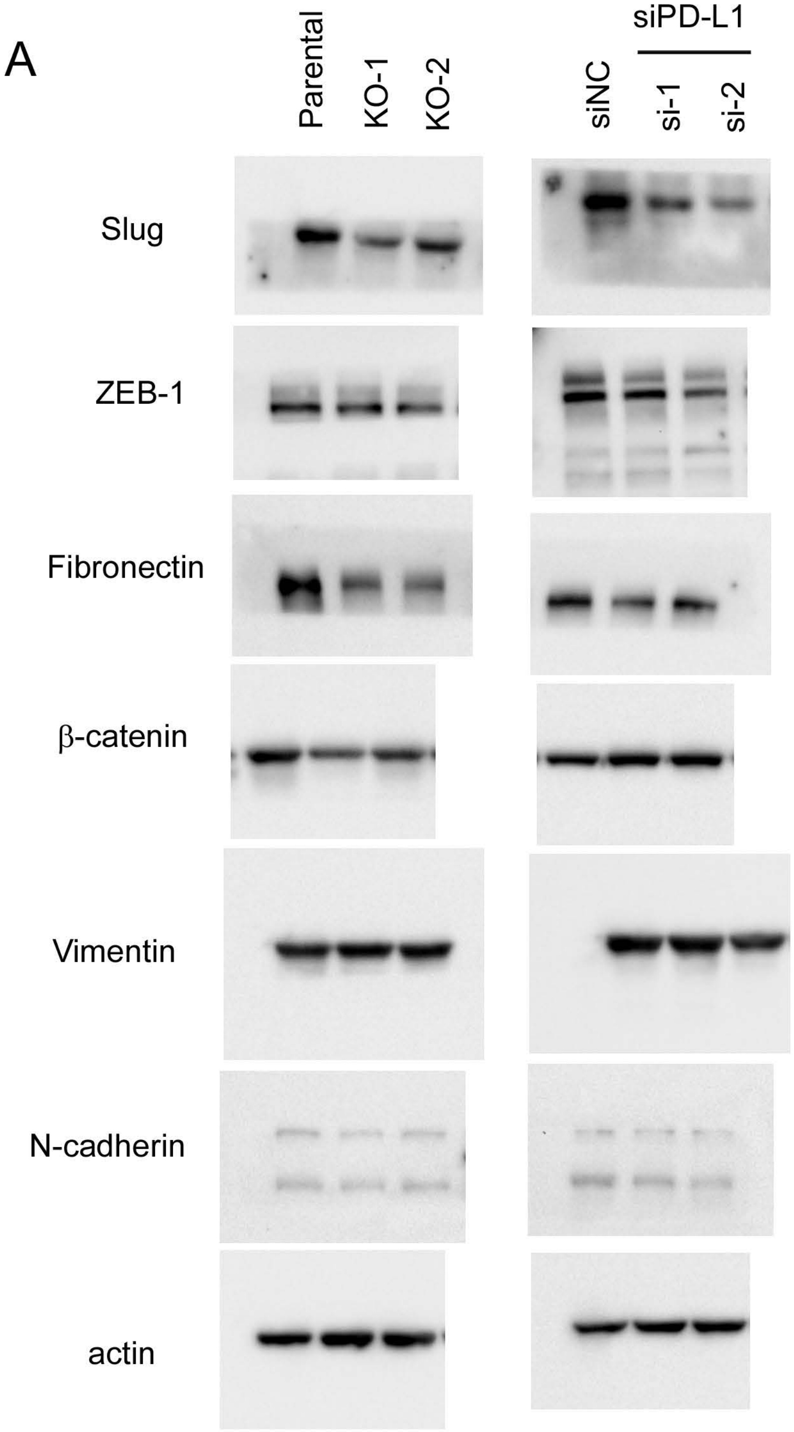

Supplementary Fig.3

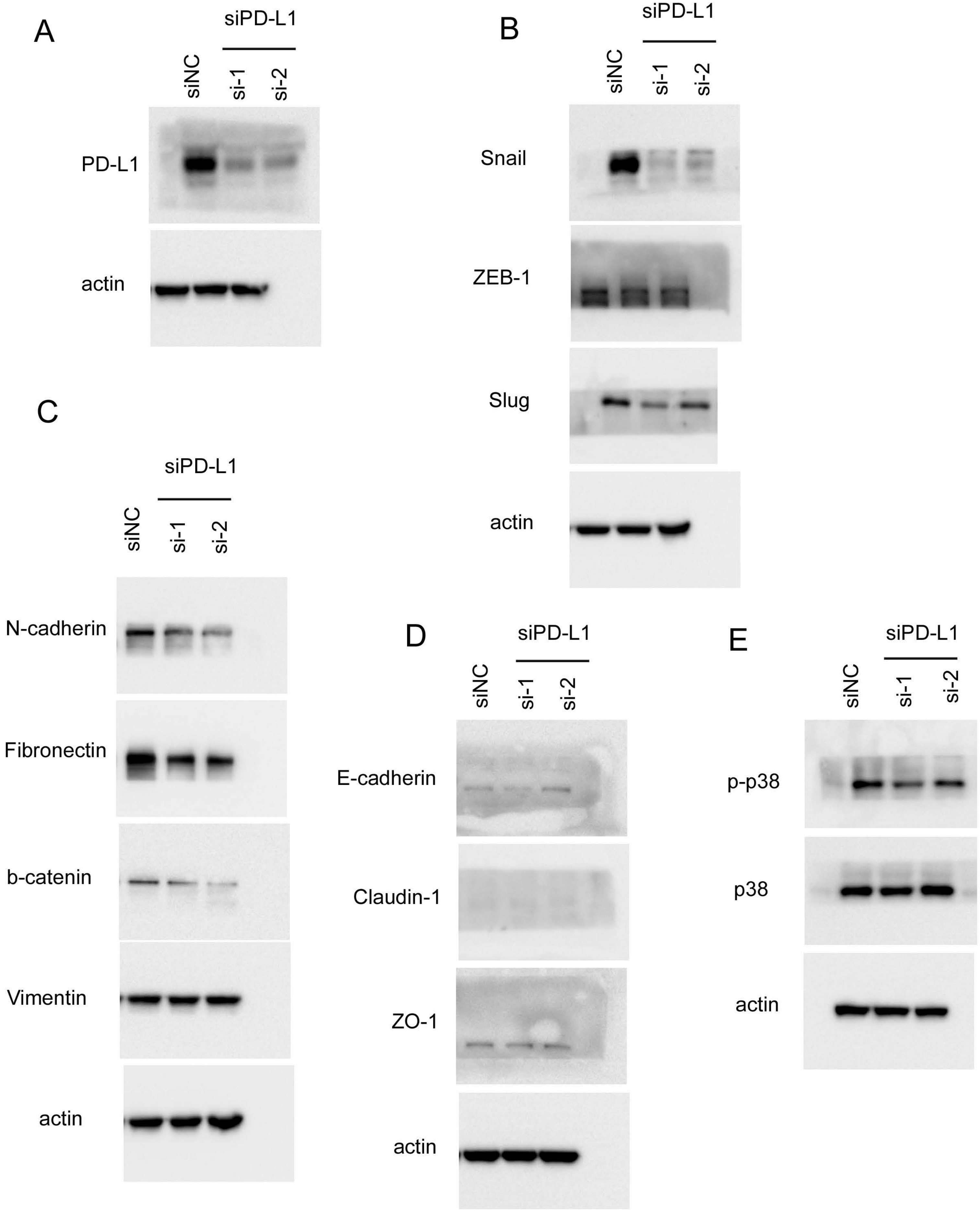

Supplementary Fig.4

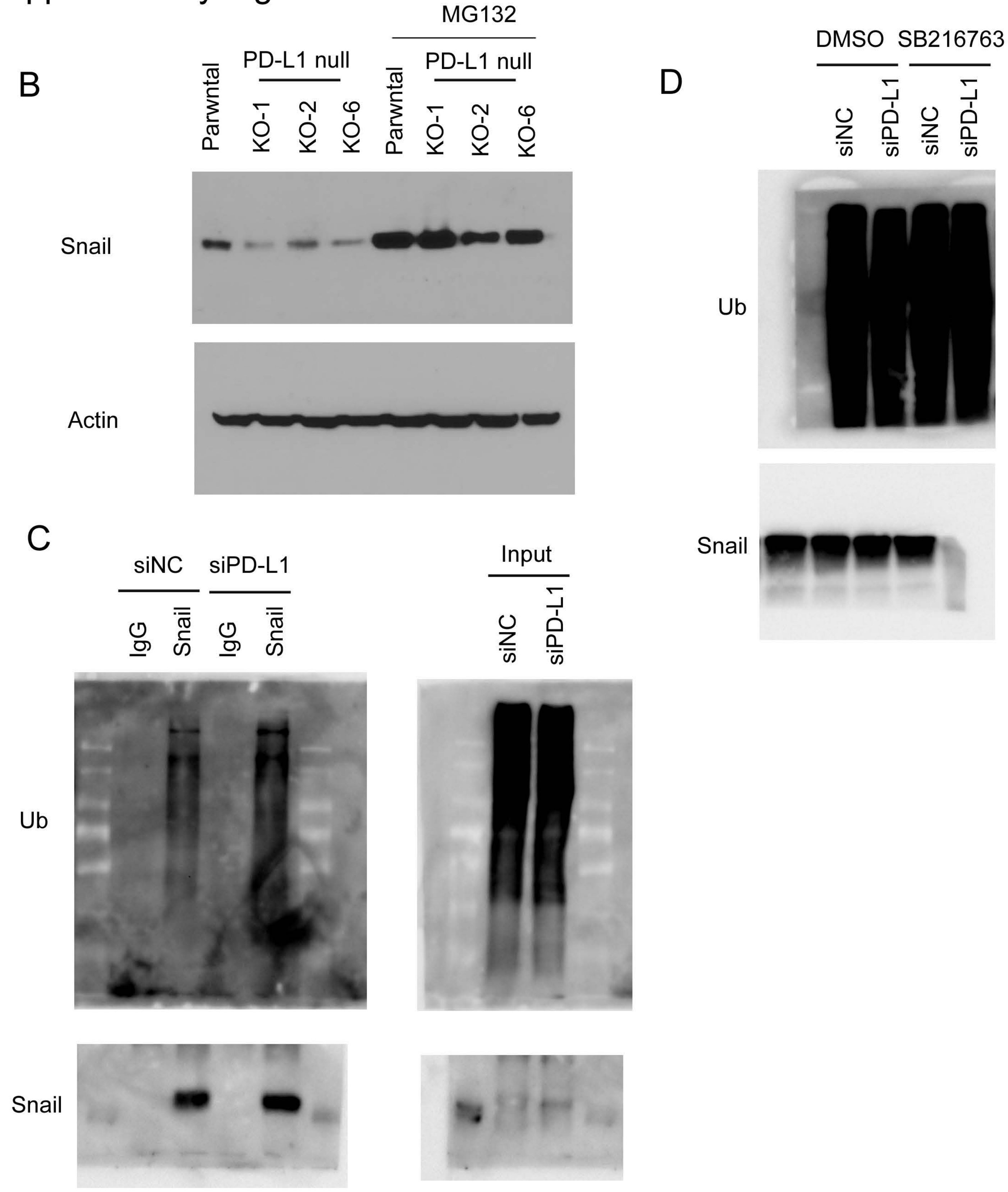

Supplementary Fig. 5

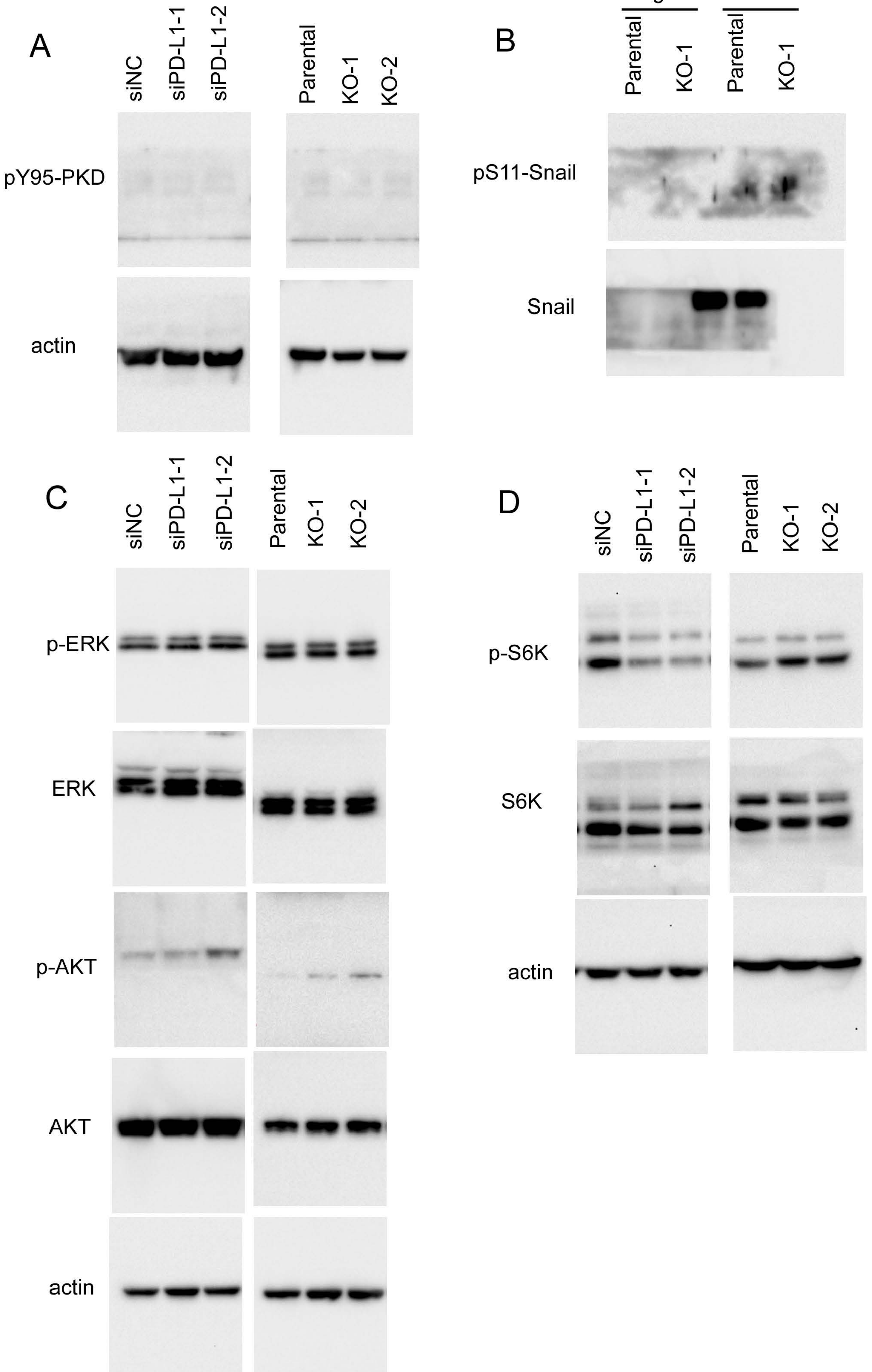

Supplementary Fig.6

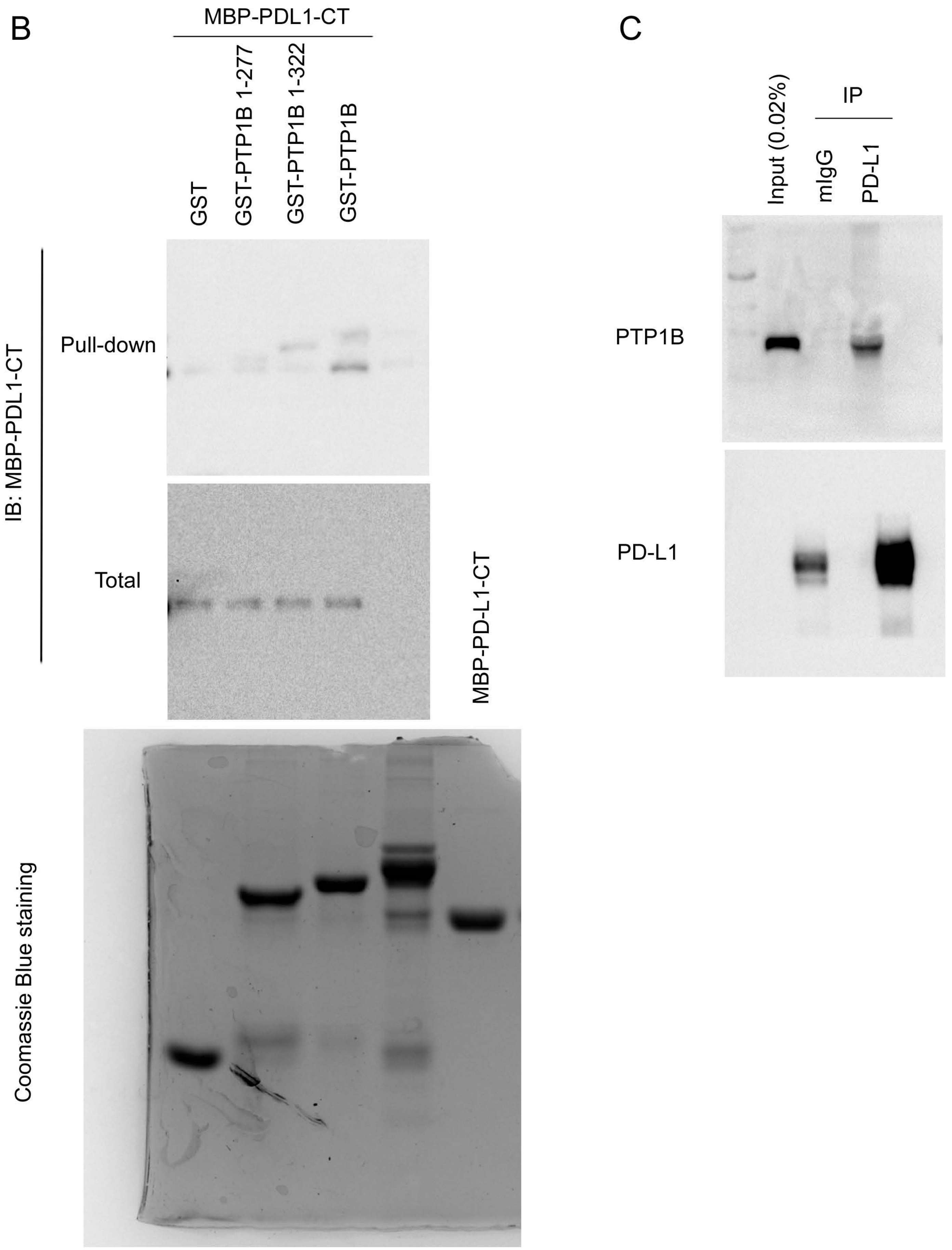

Supplementary Fig 8

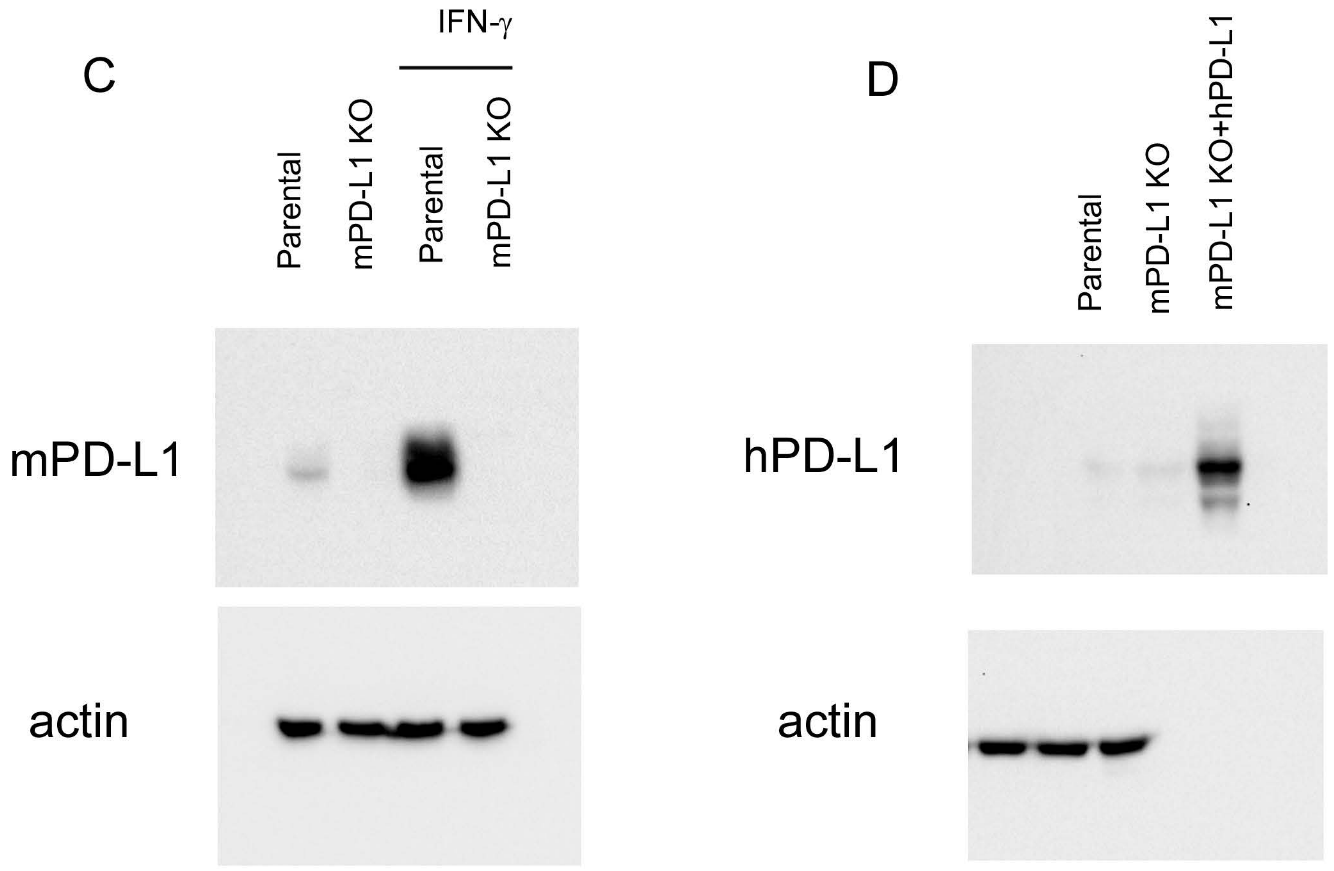

Supplement: Supplemental data [file jciinsight-6-131458-s086.pdf]
